# A comprehensive metabolomic study of three Egyptian Salsola species revealed their potential anti-inflammatory activity

**DOI:** 10.1038/s41598-024-80807-2

**Published:** 2025-02-11

**Authors:** Abdelrhman Zakaria, Fahima. F. Kassem, Doaa A. Ghareeb, Safa M. Shams Eldin, Dina A. Selim

**Affiliations:** 1 Department of Pharmacognosy, Faculty of Pharmacy, Alexandria University, Alexandria, 21521 Egypt; 2 Bio-Screening and Preclinical Trial Lab, Biochemistry Department, Faculty of Science, Alexandria University, Alexandria, Egypt; 3 Center of Excellence for Drug Preclinical Studies (CE-DPS), Pharmaceutical and Fermentation Industry Development Center, City of Scientific Research and Technological Applications (SRTA-city), New Borg El Arab, Alexandria, Egypt; 4 Research Projects Unit, Pharos University in Alexandria, Alexandria, Egypt

**Keywords:** Plant sciences, Chemistry

## Abstract

*Salsola* plants are halophytic crops that are distributed worldwide, with more than 100 species figured out in Asia, the Mediterranean region and North Africa. Different *Salsola* species were reported to exert marked anti-inflammatory activities, whereas the potential anti-inflammatory activities of the three species, *S. tetrandra*, *S. tetragona* and *S. vermiculata*, have not been evaluated. This study provides a comprehensive metabolic study of the shoots and roots of those three species to identify potential anti-inflammatory candidates. An ultra-high performance liquid chromatography mass-mass spectrometry (UHPLC MS/MS) method in conjunction with multivariate analysis principles was utilized in an attempt to decipher their bio-active metabolites and their relevant anti-inflammatory activities. Eighty metabolites were identified in the tested extracts, where nitrogenous compounds and phenolics were highly detected in *S. tetragona* samples, meanwhile, saponins and phenolic acids were highly dominant in *S. tetrendra* sample and *S. vermiculata* samples have a similar chemical profile as *S. tetrandra*. Concerning the anti-inflammatory activity of the tested extracts, the safety margin of all the tested extracts was higher than that of the standard drug piroxicam. The shoots of the three species demonstrated more potent anti-inflammatory activities compared to the roots. The shoot extract of *S. tetrandra* was the most biologically active fraction. The obtained results revealed the shoots of the three *Salosla* species to be promising anti-inflammatory drug candidates of high safety and efficacy that could be used in the pharmaceutical industry.

**Supplementary Information:**

The online version contains supplementary material available at 10.1038/s41598-024-80807-2.

## Introduction

Halophytes (salt-tolerant plants) are a group of plants that can survive in saline soils in coastal regions. They represent considerable biomass with a high degree of diversity. They represent renewable economic platforms that sustainably offer pastoral, feed, agricultural and medical supplies^1^. Yet, few research work was carried out in order to investigate halophytes^2^. Genus *Salsola*,which belongs to Chenopodiaceae or Amaranthaceae family, is a typical representative of halophytes. Being contributing to more than 100 species distributed over the world, it represents the major genus in the family. Namely; “*Salsola”* is derived from the Latin name *“Salsus”* meaning salty due to the high tolerability of these plants to saline soils. Concerning the plant ecology, *Salsola* species naturally inhabit temperate, arid and semi-arid regions, which are commonly, characterized by extremely harsh conditions. Geographically, these plants are distributed in central and southwestern Asia, the Mediterranean region and North Africa. That genus is cosmopolitan, where diversity was observed in its species ranging from perennial herbs to annuals, sub-shrubs, shrubs and small trees. Unfortunately, discrimination between different species of *Salsola* is challenging because of their close morphological similarities^2,3^. Moreover, fourteen species of the investigated genus are endemic to Egypt, located on the Mediterranean Coast, Sinai and the Red Sea region^4^.

Previous phytochemical investigations of *Salsola* revealed the tremendous diversity in their matrices. Previous phytochemical investigations of different Salsola species exhibited that flavonoids, nitrogenous compounds, phenolic compounds and triterpenoids were abundant chemical classes in these extracts^2,3,5^. Furthermore, the biological screening of some *Salsola* plants has demonstrated profound activities as hypoglycemic, antioxidant, anti-microbial, cytotoxic and anti-inflammatory putative^5–9^. In the same context, the protective activities of these extracts on the cardiovascular, neurological and digestive systems were recorded^3,8^.

A wide range of research work effectively clarifies the anti-inflammatory activity of crude extracts and phytochemicals, exhibiting their ability to relieve different conditions of inflammation ranging from being localized to a generalized condition and from being an acute problem to a chronic matter. They also represent drugs and lead compounds for designing safe and effective anti-inflammatory agents, devoid of the serious side effects of existing synthetic molecules with anti-inflammatory potential^10^.

*Salsola* is extensively consumed in folk medicine to treat various stages of inflammatory conditions, targeting the skin, the digestive system and the respiratory system^3^. Most *Salsola* species exhibited auspicious results when testing their anti-inflammatory activity; considering the in-vitro studies, *S. soda* markedly inhibited aldose reductase enzyme, an enzyme responsible for the synthesis of pro-inflammatory mediators^6^. Moreover, the butanol extract of *S. imbricta* inhibited the release of nitric oxide, known to largely contribute to tissue injury. As well as *S. komarovii* extract, it could reduce mucosal damage in an ulcerative colitis simulating model. Whereas in-vivo studies revealed that *S. cycophylla* and *S. imbricta* extracts reduced edema, pain and inflammatory conditions in experimental animals^3,9,11,12^.

Despite the marked antioxidant activity of *S. tetrandra* and *S. vermiculata*, up to our knowledge, no studies for their potential anti-inflammatory activities, as well as the biological activity of *S. tetragona*, have been screened yet. Moreover, no previous study was able to provide a comprehensive chemical profile of these three plants. Hence, our work aimed to provide a comprehensive metabolomic study of these three Egyptian species for the first time. To fulfill this objective, an ultra-high performance liquid chromatography-mass-mass (UHPLC MS/MS) analysis was implemented in conjunction with multivariate analysis. The potential anti-inflammatory activity of the tested samples was screened to correlate the chemical metabolomic profiles of the tested extracts to their biological matrices, revealing the most biologically active fraction that could be a promising safe and effective anti-inflammatory candidate.

## Methods

### Plant material

Three species of *Salsola* were investigated in this work, namely, *Salsola tetrandra* Forssek, *Salsola vermiculata* L and *Salsola tetragona* Delile.

The samples were collected from Al-Amaya Zawyt Abdelkader and Burg Al-Arab in August 2021, during the vegetative periods of the three species. All samples were authenticated by Prof. Dr. Sania Ahmed, Professor of Plant Ecology, Faculty of Science, Alexandria University, through comparable identification with authentic samples allocated at the Alexandria University Herbarium. Samples of the collected species were deposited in the same herbarium, voucher numbers: Sal. tetrandra 123, Sal. tetragona 124 and Sal. vermiculata 125.

### Sample preparation

Shoots were separated from the roots for each species and dried in the shed at 20 °C. Fifty grams of each shoot and root part from the three *Salsola* species were independently ground using an electric grinder and macerated in 70% ethanol for 14 days. We performed a pilot study to find out the optimum conditions for extracting the three species of *Salsola*, including extracting them for 24 h, 3 days, 7 days and 14 days as mentioned in the previous work^2,13,14^. The maximum number of metabolites was obtained after 14 days of maceration, as described by^13^.

The hydro-alcoholic extracts were dried under vacuum using Buchi rotavap RT 210 rotary evaporator^13^.

### Sample and authentic compound preparation for UHPLC-MS/MS analysis

Dilution of each extract of the three species in HPLC-grade methanol was performed followed by filtration through a membrane disc filter of 0.2 μm pore size, followed by degassing by ultrasonic sonication and finally, 10 µL of the sample volume was loaded at a concentration of 1 mg/ml on the reversed-phase column in the full loop mode injection. The injection process of each sample occurred in five replicates on 5 consecutive days to ensure the reproducibility of the method^15^. Standardization, repeatability and stability of the analytical work were guaranteed by pooling a 5µL aliquot of each sample as a quality control sample that could be used as authentic data to be extracted, as it had all the sample information^16^.

For the authentic compounds, The procedure was performed as follows: Dilution of each extract in HPLC-grade was in methanol followed by filtration through a membrane disc filter of 0.2 m pore size, then degassing by sonication, and finally, 10 µL of the sample volume was loaded at a concentration of 1 mg/ml on the reversed-phase column in the full loop mode injection. The injection process for each sample occurred in five replicates.

### The conditions of ultra-high performance liquid chromatography utilized to chemically profile *Salsola* species

To identify the *Salsola* metabolites in different extracts, a Shimadzu 8045 UPLC triple quadrupole (TQD) instrument composed of a pump (LC-2040), an autosampler (LC-2040), a detector (LC-2030/2040 PDA detector), a degasser and a Shimpak UPLC C18 column of 1.7 μm particle size, 2.1 mm internal diameter and 150 mm length was used. The pump worked in a low-pressure gradient mode with a flow rate of 0.2 ml/min and a maximum pressure of 66 MPa in auto-LPGE mode. The autosampler’s rinsing volume, rinsing speed, sampling speed, purge time and cooler temperature were 500 µL, 35 µL/s, 5 µL/s, 2 min and 15 °C, respectively. In addition, the rinse mode was repeated before and after aspiration. The photodiode array detector (PDA) was operated by a D2 lamp covering a wavelength range from 200 to 800 nm. The cell temperature was 40 °C and the slit width was 8 nm, which was used in both positive and negative modes. Channels 1 and 2 wavelengths, bandwidths and output ranges were 254 nm, 4 nm and 1.0 AU/V, respectively. The mobile phase system was ultra-pure water (Phase A) and HPLC-grade acetonitrile (Phase B) and the total chromatographic run lasted for 35 min. The elution gradient was set as the following: 10% of B at the retention time of 0.01–5.00 min, 30% of B at 5.00–15.00 min, 70% of B at 15.00–22.00, 80% of B at 22.00–26.00 and 10% of B at 29.00–30.00.

### The conditions of electro-spray ionization-mass spectrometry (ESI-MS)

A mass spectrometer with a (TQD) coupled to an (ESI) source was used to analyze *Salsola* metabolites in both positive and negative modes. A TQD mass analyzer was used for further fragmentation of the ions, producing daughter ion fragments that facilitate the identification process. The first and third quadrupoles act as mass filters, meanwhile, the second one acts as a collision-induced dissociation cell^15,17^.

ESI conditions were 2.51 L/min nebulizing gas flow, 10.00 L/min drying gas flow, 300 °C interface temperature and 526 °C de-solvation temperature. The conversion dynode was 2.00 kV, the acquisition mass range was from m/z 100.00 to m/z 1500.00, which is the auto-range, the scan speed was 1428 u/s, the interface volt was 4.00Kv, the DUIS corona needle volt was 4.50 kV, the acquisition mode was Q3 scan in both segment 1 Event 1 and segment 1 Event 2, the capillary’s voltage was 3 kv, the cone’s voltage was 35 v, the ion source’s temperature was 150 °C and the nitrogen gas nebulizer’s pressure was 35 psi.

It is worth mentioning that six external standards, namely, caffeic acid, hesperidin, scopoletin glucoside, tyramine, azalaic acid and oleanolic acid (Sigma-Aldrich Co., St. Louis, MO, USA)], were used to construct calibration curves expressed in (mg standard equivalent/g) dry extract using an exact weight of the reference (10 mg) was dissolved in 10 mL of HPLC- grade methanol to create each reference solution. For generation of calibration curves from reference compounds, five µLs from each concentration level were injected onto the HPLC column in duplicate after the solution had been serially diluted to working concentrations. Those curves obtained from the reference compounds provided a tool for peak area calculations of all detected metabolites according to their chemical class. Plotting the peak area of each standard concentration on the Y-axis against the known concentration on the X-axis was performed to construct the calibration curve of each standard. The limit of quantitation (LOQ) and the limit of detection (LOD) parameters were detected to ensure method validation. Furthermore, all parameters were assessed based on the FDA guidelines on bioanalytical method validation^15^. Standard preparation and calibration curve validation are discussed in Table 1. The regression equation is y = ax + b, where y is the peak area, x is the standard concentration in mg/ml, a is the slope, and b is the intercept.

**Table 1:** Linearity and sensitivity parameters for caffeic acid, hesperidin, tyramine, scopoletin glucoside, azalaic acid and oleanolic acid.

Compound	Linearity range (mg/L)	Slope (a)	Intercept (b)	Correlation coefficient	LOD (mg/L)	LOQ (mg/L)
Caffeic acid	0.1–10	0.03300	0.0036	0.992	0.028	0.093
Hesperidin	5–50	0.018	−0.003	0.999	0.139	0.24
Scopoletin glucoside	12–1500	1.0325	−0.0057	0.9995	0.9	29
Tyramine	3–10	0.1973	0.0274	0.9996	0.05	0.25
Oleanolic acid	24.4–294	1.1558	5.0379	0.996	8.6	28.8
Azalaic acid	0.1–10	17.457	954.97	0.9929	0.18	0.54

### Determination of cytotoxicity and anti-inflammatory activities of *Salsola* species

3-(4,5-dimethylthiazol-2-yl)-2,5-diphenyl-2 H-tetrazolium bromide (MTT) assay was carried out to establish the cytotoxicity of all *Salsola* extracts and their results were compared to those of standard piroxicam. The effective anti-inflammatory concentrations (EAICs) were estimated for each fraction extract in lipopolysaccharide (LPS)-stimulated human white blood cell culture. The expression levels of genes coding pro-inflammatory mediators IL-1β, IL6, TNF-α and TNF-γ were determined by real-time polymerase chain reaction (PCR). Results were expressed as means ± standard deviation (SD) of three individual replicates.

#### Isolation and cultivation techniques of human white blood cells WBCs

To prevent coagulation, the obtained human blood specimen was kept in a heparin tube. In a 15 ml centrifuge tube, a 1 ml blood specimen was treated with a fresh lysing solution to get rid of red blood cells. The lysing solution filled the tube to capacity. Then, the centrifuge tube was inverted for about 10 min at room temperature until the liquid became clear red. This was followed by centrifugation of lysed blood specimen at 4 °C, 2000 rounds per minute (rpm) for 10 min, decantation of the supernatant, and then drainage of the tube. In 10 ml cold phosphate buffered solution (PBS), the WBCs were suspended, then centrifugated. The isolated WBCs were re-suspended in Roswell Park Memorial Institute Medium (PRMI).

Viability and WBCs count were assessed by Dye Exclusion Method by mixing 50 µL of cell suspension with an equal volume of 0.5% trypan blue stain solution. After that, the mix was loaded onto a hemocytometer. Only the non-viable cells stained and counting process occurred in the four corner quadrants (A, B, C, and D).

$$\text{N/ml}= \text{mean of WBCs counting}\times10^{4}\times \text{D}.$$

N is the number of viable or non-viable cells, and D is the sample dilution (1:1 trypan blue solution).

$$\% \text{ Cell viability}=\text{no of viable cells/total no of cells}\times 100.$$

Only mediums containing at least 90% viable cells were used for further investigations. The incubation conditions of the RPMI culture medium were 37 °C temperature, 5% carbon dioxide, and 90% relative humidity for six days in a carbon dioxide incubator. The cell titer was 100.000 cells/well in a 96-well plate.

#### Cytotoxicity assessment of the crude extracts compared to standard piroxicam

200 µL of cultured medium containing 100.000 WBCs/well were treated with different concentrations (0, 3.125, 6.25, 12.5, 25, and 50 µg/ml) of the crude extracts in an RPMI medium without bovine fetal serum or piroxicam (standard anti-inflammatory drug), then the plates were incubated at 37 °C temperature, 5% CO_2_, and 90% relative humidity for 72 h in a CO_2_ incubator.

After three days of incubation, 20 µL of MMT solution was added to each well followed by plate incubation to allow the progress of the MTT reaction. This was followed by centrifuging the incubated medium at 1650 rpm for 10 min and discarding the medium. MMT byproducts (formazan needles) were re-suspended in 100µL dimethyl formamide (DMSO). The absorbance of re-suspended crystals was measured at 570 nm using a spectrophotometer to detect a safe dose, which causes 100% viability.

The mathematical equation for calculation of % cell viability:

$$\text{A}_{\text{T}}=\text{A}_{\text{b}}/{\text{A}}_{\text{c}}-\text{A}_{\text{b}}\times 100.$$

AT = mean absorbance of cells treated with different concentrations of each crude extract. Ac = mean absorbance of untreated cells with culture medium only. Ab = mean absorbance of cells treated with the vehicle of plant extract (PRMI without fetal bovine serum).

The cytotoxicity assay of the compound expressed as EC_100_ was calculated by the Graphed Instat software using % viable calculated from the serial dilutions of each plant extract.

#### Determination of the effective anti-inflammatory concentration (EAICs) of the crude extracts in lipopolysaccharides (LPS) stimulated human WBCs culture

In a 96-well plate, a volume of 50 µL of the culture medium containing 100,000 human WBCs was dispensed per well. Induction of inflammation occurred by adding 50 µL of LPS to the plated cells and incubation them in a CO_2_ incubator for 24 h. This was followed by centrifugation at 1650 rpm for 5 min, then discarding of the supernatant, and adding 200 µL of different concentrations (0,3.125, 6.25, 12.5, 25, and 50 µg/ml) of crude extracts and piroxicam.

The control cells (which were the negative control) containing the culture medium only and the tested plates were incubi ted for 72 h in the CO_2_ incubator.

The proliferation of the stimulated cells was determined using an MTT assay (please refer to the previous section dealing with the steps of the MTT assay).

Cell proliferation was assessed using the stimulation index (SI), which is the mean absorbance of LPS-stimulated WBCs treated with different concentrations of crude extracts/absorbance of control untreated cells.

EAIC of each crude extract is the ability to retrieve the abnormal proliferation of LPS-stimulated cells to the normal proliferation of un-stimulated cells. SI = 1 was calculated using an Instate graph pad.

#### Extraction of RNA of untreated and treated LPS stimulated human white blood cells

Extraction of RNA started with re-suspension of cell pellets in 50 µL solution R1, mixing for 30 s, then incubation at room temperature for 1 min. This was followed by adding 300 µL of solution R2 and incubation at 4 °C for 3–5 min. The obtained supernatant was loaded on a spin column and centrifuged for 30 s at 4 °C, 14.000 rpm. After that, 300 µL of working wash buffer was added to the spin column and the flow-through was discarded. Centrifugation was done for 30 s, and the described process was repeated twice.

Another centrifugation for 1 min at 10.000 rpm was done and transferring the centrifuge into a sterile 1.50 ml microcentrifuge tube was followed. To the center of the membrane, 30µL of elution buffer was added and incubated at room temperature for 1 min, then centrifugated for 30 s at 14.000 rpm, 4 °C.

The optical density of the extracted RNA was determined by mixing the absorbance and purity at A_260_, and A_260_/A_280_ nm respectively using a spectrophotometer and kept at −80 °C until real-time polymerase chain reaction (PCR).

#### cDNA synthesis from RNA extracted from untreated and treated LPS stimulated white blood cells

In a PCR tube, 2 µg of total RNA or nuclease-free water and 1 µg of oligo dT primer were added to the nuclease-free water to obtain a total of 12 µL. This was followed by gentle mixing, centrifugation, incubation at 65 °C for 5 min in a PCR machine, and placing back on ice immediately.

The gentle mixing of 4 µl of 5× reaction buffer, 1 µl of RNase inhibitor, 2 µl of the dNTPs mix, and 1 µl of reverse transcriptase or 1 µl of nuclease-free water instead of reverse-transcriptase for reverse transcriptase negative control with the previous mixture was performed. After that, spin down and incubation for 60 min at 42 °C followed by heat inactivation at 70 °C for 5 min in the PCR machine was carried out.

#### Determination of IL-1β, IL 6, TNF, and INF-γ expression levels by real-time polymerase chain reaction (PCR)

30 µl of 2× SYBR green master mix was mixed with 5 µl of cDNA, 0.5 µl of 10 pmoles/ml forward primer, and 0.5 µl of pmoles/ml reverse primer for each primer in PCR tubes. As for the reference tube, 0.5 µl of 10 pmoles/ml forward primer of β-actin and 0.5 µl of 10 pmoles/ml for reverse primer of β- actin were added. To assess for reagent contamination or primer dimers, another tube was used as a non-template control (NTC) by adding 1 µl of nuclease-free water instead of a template used. After that, the gentle mixing of the tubes with 6.5 µl nuclease-free water without creating bubbles was done and then spun for a few seconds. In the cycler, samples were placed, and the program was started as the following: initial denaturation (1 cycle of 95 °C for 10 min), followed by denaturation (40 cycles of 95 °C for 15 s), annealing (at 60 °C for 30s), and extension (at 72 °C for 30 s). the effect of LPS and extracts on gene expression was expressed as a Fold change in gene expression which was calculated according to the following equations:

**Calculation**

- **Expressions fold levels of gene calculated by**
  - ΔCt _normal_ = Ct _normal untreated cells_ – Ct _reference_
  - ΔCt _tested plant extract_ = Ct _tested plant extract−treated cells_ – Ct _reference_
  - ΔCt _induced_ = − Ct _LPS−exposed cells_ – Ct _reference_
- **In case of genes**:
  - ΔΔCT_tested plant extract_ = ΔCt _tested plant extract_ – ΔCt _normal_
  - ΔΔCT_induced_ = ΔCt _induced_ – ΔCt _normal_
- **In case of GAPDH**:
  - ΔΔCT_tested plant extract_ = ΔCt _normal_ – ΔCt _tested plant extract_
- ΔΔCT_induced_ = ΔCt _normal_ – ΔCt _induced_

$$\text{Fold change in gene expression}=\text{log}^{ (2-\Delta \Delta CT)}$$

Where: Ct tested plant extract: threshold cycle value of genes of extracted mRNA of plant extract treated-LPS-stimulated WBCs which is defined as the cycle number at which the fluorescence generated within a reaction crosses the fluorescence threshold. Ct reference: threshold cycle value of GAPDH which is used for normalization. Ct normal: threshold cycle value of genes of extracted mRNA of untreated control WBCs. Ct induced: threshold cycle value of gene of extracted mRNA of LPS-stimulated WBCs.

The primers used:

**Table:** 

TNF-*α*	F-CTCTTC TGCCTGCTGCACTTTG
	R- ATGGG CTACAGGCTTGTCACTC
IL-6	F, 5′-TGAA CTCCTTCTCCACAAGCG-3′
	R, 5′-TCTG AAGAGGTGAGTGGCTGTC-3′
IL-1*β*	F, CCACA GACCTTCCAGGAGAATG
	R, GTGCA GTTCAGTGATCGTACAGG
INF-γ	F, GAGTG TGGAGACCATCAAGGAAG
	R, TGCTT TGCGTTGGACATTCAAGTC
	R, GGAAGATGGTGATGGGATT
GAPDH	F, GGATTTGGTCGTATTGGG
	R, GGAAGATGGTGATGGGATT

### Statistical analysis

One-way analysis of variance (ANOVA) is the statistical tool that was used for semi-quantitative analysis and anti-inflammatory activity investigation. The peak intensities of the identified metabolites were used to calculate the relative concentration of each metabolite as mg equivalent (Eq.)/g dry weight dry extract and the data of the retrieving power of each extract to bring the LPS-stimulated WBCs to the normal state of each extract was used to calculate the effective anti-inflammatory concentration of each one. Multivariate analysis of the metabolomics data was performed using SIMCA 14 software (Umetrics, Malmo, Sweden). The heat map was constructed using Metaboanalyst 5.0 (http://www.metaboanalyst.cal/), an online free tool for data analysis.

The relative concentrations obtained from semi-quantitative analysis were used to construct the heat map.

## Results and discussion

### Metabolite annotation of different *Salsola* extracts

Six *Salsola* samples, namely, shoot and root 70% ethanolic extracts of *S. tetrandra*, *S. vermiculata* and *S. tetragona*, were subjected to UHPLC-MS/MS analysis to identify their principle active constituents. Eighty compounds were tentatively identified in all extracts of the aforementioned three *Salsola* species through investigating both positive and negative ESI-MS modes, where they included phenolic compounds, flavonoids, triterpenes, saponins, coumarins, fatty acids and nitrogenous compounds in the negative and positive ion modes (Table 2, Fig. S1). The compounds were assigned in accordance with their retention times, recorded MS/MS fragment ions and fragmentation patterns as compared to an in-house database with about 300 metabolites of varying chemical classes from the entire *Salsola* genus, as well as Mass bank database reference and the Dictionary of Natural Products. The in-house database composed only metabolites identified in *Salsola* species, ignoring any other species. The total number of compounds in the library was 312 compounds of all identified chemical classes in genus *Salsola*. During constructing the in-house library, we selected only compounds that were identified in different *Salsola* species using LC-MS technique, where ESI conditions were applied, the reversed-phase column C18 was used and the same composition of the mobile phase or even the same polarity and the same gradient elution mode The elution order of the metabolites was also a main factor in the compound elucidation.

**Table 2:** Identified metabolites in Salsola extracts using UPLC MS/MS analysis in both negative and positive modes.

No	RT	m/z	Identified compound	Precursor ion	Molecular formula	Molecular weight	Chemical class	MS^2^	References
1	0.67	365.234	Trehalose	[M+Na]^+^	C_12_H_22_O_11_	342.3	Disaccharide	302	^60^
2	0.7314	193.113	D-galacturonic acid	[M−H]^−^	C_6_H_10_O_7_	194.1	Sugar acid	131, 59	^61^
3	0.7788	118.103	Betaine	[M+H]^+^	C_5_H_11_NO_2_	118.1	Amino acid, glycine derivative	58	^53^
4	2.202625	153.174	Hypogallic acid	[M−H]^−^	C_7_H_6_O_4_	154.1	Phenolic acid	109	^49^
5	2.6465	180.100	Pericampylinone-A	[M+H]^+^	C_9_H_9_NO_3_	179.2	Nitrogenous compound	192, 178, 149	^13^
6	2.7135	385.050	Cleomiscosin B	[M−H]^−^	C_20_H_18_O_8_	386.4	Coumarinolignoid	367, 207, 179,136, 123	^13^
7	3.16575	167.199	Orsellic acid	[M−H]^−^	C_8_H_8_O_4_	168.2	Phenolic acid	149, 123, 105	^48^
8	3.33325	179.100	caffeic acid	[M−H]^−^	C_9_H_8_O_4_	180.2	Phenolic acid	161, 135, 107	^47^
9	4.556	153.100	Anisic acid	[M+H]^+^	C_8_H_8_O_3_	152.3	Phenolic acid	135, 109, 94, 92, 77, 66, 51, 45	^51^
10	5.91275	593.400	Dirhamnosyl quercetin	[M−H]^−^	C_27_H_30_O_15_	594.5	Flavonoid	473, 447, 301, 299	^21^
11	6.14725	359.200	Biphenyl salsinol	[M+H]^+^	C_20_H_22_O_6_	358.4	Phenolic compound	381 [M+Na]	^73^
12	6.29241	330.166	N-(4’-methoxcinnamoyl)-norepinephrine	[M+H]^+^	C_18_H_19_NO_5_	329.4	Nitrogenous compound, cinnamide	170, 153, 107	^13^
13	6.31475	183.199	O-methyl gallic acid	[M−H]^−^	C_8_H_8_O_5_	184.1	Phenolic acid	168, 140, 124	^50^
14	6.9019	330.141	Feruloyl octopamine	[M+H]^+^	C_18_H_19_NO_5_	329.4	Nitrogenous compound, cinnamide	154, 136	^13^
15	6.9143	312.060	Taxiphyllin	[M+H]^+^	C_14_H_17_NO_7_	311.3	Cyanogenetic glycoside	179	^13^
16	6.928	328.083	Hydroxy moupinamide	[M−H]^−^	C_18_H_19_NO_5_	329.4	Nitrogenous compound, tyramine derivative	194, 136	^2^
17	6.98475	609.250	Glucosyl rhamnosyl -quercetin	[M−H]^−^	C_27_H_30_O_16_	610.5	Flavonoid	608, 463, 447, 301, 299	^21^
18	7.110	342.087	Hernandine	[M+H]^+^	C_19_H_19_NO_5_	341.4	Nitrogenous compound, quinoline derivative	327, 296, 268	^54^
19	7.400	298.108	N-caffeoyl tyramine	[M−H]^−^	C_17_H_17_NO_4_	299.3	Nitrogenous compound, tyramine derivative	209, 178, 161, 136	^2^
20	7.545	187.179	Azelaic acid	[M−H]^−^	C_9_H_16_O_4_	188.2	Dicarboxylic acid	169, 143, 125	^55^
21	7.822	312.100	N-feruloyl tyramine	[M−H]^−^	C_18_H_19_NO_4_	313.3	Nitrogenous compound, tyramine derivative	297, 279, 178, 136	^2^
22	7.906	342.100	N-feruloyl-3′′′-methoxy Tyramine	[M−H]^−^	C_19_H_21_NO_5_	343.3	Nitrogenous compound, tyramine derivative	326, 193, 136	^2^
23	8.224	405.150	Biphenyl salsonoid B	[M+H]^+^	C_21_H_24_O_8_	404.4	Phenolic compound, biphenyl propanoid	153	^13^
24	8.525	609.233	Hesperidin	[M−H]^−^	C_28_H_34_O_15_	610.6	Flavonoid	447, 301, 286, 258, 257, 244, 242, 151	^19^
25	8.626	301.220	Tachioside	[M−H]^−^	C_13_H_18_O_8_	302.3	Phenolic acid glycoside	139	^13,43^
26	8.626	551.250	Salsotetragonin	[M−H]^−^	C_29_H_44_O_10_	552.7	Cardenolide	389	^13^
27	9.162	619.251	Glucopyranosyl oleanolic acid	[M+H]^+^	C_36_H_58_O_8_	618.8	Triterpenoid	457, 439, 411, 393	^40^
28	9.195	609.311	Diosmin	[M+H]^+^	C_28_H_32_O_15_	608.5	Flavonoid	447, 301, 191	^20^
29	9.497	441.299	Salsamine	[M+H]^+^	C_26_H_36_N_2_O_4_	440.5	Nitrogenous compound, isoquinoline alkaloid	192, 178, 149	^13^
30	9.524	941.434	Salsoloside D	[M−H]^−^	C_47_H_74_O_19_	942.5	Saponin glycoside	779, 647, 471	^6,59^
31	9.983	1087.501	Salsoloside E	[M−H]^−^	C_53_H_84_O_23_	1088.5	Saponin glycoside	925, 763, 631, 455	^6^
32	10.05	681.502	Pentahydroxy- oleanen-oic acid glucopyranoside	[M−H]^−^	C_36_H_58_O_12_	618.5	Triterpene saponin glycoside	519, 471, 455, 441, 427	^13^
33	10.083	353.2 11	Daphnoretin	[M+H]^+^	C_19_H_12_O_7_	352.3	Coumarin derivative	336, 191	^32^
34	10.318	164.122	p-Coumaramide	[M+H]^+^	C_9_H_9_NO_2_	163.2	Phenolic amide	146	^45^
35	12.746	925.400	Salsoloside C	[M−H]^−^	C_47_H_74_O_18_	926.5	Saponin glycoside	763, 631, 455	^6^
36	12.914	505.299	Salsolin A	[M+H]^+^	C_30_H_48_O_6_	504	Triterpenoid	459, 224, 280	^39^
37	13.132	203.102	Bergaptol	[M+H]^+^	C_11_H_6_O_4_	202.2	Furanocoumarin, 5-hydroxy psoralen	175, 162, 147, 116	^13^
38	13.232	291.202	Catechin	[M+H]^+^	C_15_H_14_O_6_	290.3	Flavonoid	273, 139	^22^
39	13.333	243.199	Uridine	[M−H]^−^	C_9_H_12_N_2_O_6_	244.2	Nitrogenous compound, glycosylated pyrimidine analog	111	^52^
40	13.5005	359.25	Rosmarinic acid	[M−H]^−^	C_18_H_16_O_8_	360.3	Phenolic compound	197, 179, 161	^42^
41	13.634	209.123	Fraxetin	[M+H]^+^	C_10_H_8_O5	208.2	Coumarin derivative (Methoxylated coumarin)	194, 166	^34^
42	14.388	357.299	Trimethoxy methylene dioxy isoflavone	[M+H]^+^	C_19_H_16_O_7_	356.3	Isoflavonoid	342, 341, 327, 312, 195, 162	^29^
43	14.511	384.299	Trihydroxy decosan trienoic acid	[M−H^]−^	C_22_H_41_O_5_	385	Trihydroxy unsaturated fatty acid	366, 348, 330	^2^
44	14.714	285.225	Kaempferol	[M−H]^−^	C_15_H_10_O_6_	286.2	Flavonoid	257, 151	^23^
45	14.873	315.273	Phenylethyl -gluco- Pyranoside	[M−H]^−^	C_14_H_20_O_8_	316.3	Phenolic compound	153	^44^
46	14.907	223.145	Isofraxidin	[M+H]^+^	C_11_H_10_O_5_	222.1	Coumarin derivative, hydroxy coumarin	208	^35^
47	14.957	443.299	olean–en-diol	[M+H]^+^	C_30_H_50_O_2_	442	Triterpenoid	425, 423	^40^
48	14.991	353.299	Scopoletin glucoside	[M−H]^−^	C_16_H_18_O_9_	354.3	Coumarin glycoside	191, 179, 176, 173, 149, 135	^36^
49	15.259	273.299	Naringenin	[M+H]^+^	C_15_H_12_O_5_	272.2	Flavonoid	153, 147	^24^
50	15.292	421.299	Salsolains A	[M−H]^−^	C_21_H_26_O_9_	422.4	Lignan	445 [M+Na]^+^	^74^
51	15.415	489.299	Salsolic acid	[M+H]^+^	C_30_H_48_O_5_	488.7	Triterpenoid	471, 433, 425	^40,41^
52	15.493	387.299	Blumenyl glucopyranoside	[M−H]^−^	C_19_H_32_O_8_	388.5	Megastigmane glycoside	441 [M+Na]	^68^
53	15.795	551.349	Cuneataside C	[M−H]^−^	C_19_H_28_O_12_	448.4	Phenolic glycoside	389, 343	^13^
54	385.299	385.299	Staphylinoside D	[M−H]^−^	C_19_H_30_O_8_	386.4	Megastigmane glycoside	385 [M−H]^−^	^68^
55	15.845	263.223	Hydroxy pseudoguai en-olide; didehydro ketone	[M+H]^+^	C_15_H_18_O_4_	262.3	Sesquiterpene lactone	244, 229, 216, 201	^65^
56	16.214	433.299	Canthoside D	[M−H]^−^	C_18_H_26_O_12_	434.1	Phenolic glycoside	271	^13^
57	16.465	425.299	Lupeol	[M−H]^−^	C_30_H_50_O	426.7	Triterpenoid	407, 363, 300, 288, 256, 244, 214	^38^
58	16.632	293.150	Gengirol	[M−H]^−^	C_17_H_26_O_4_	294.4	Phenolic compound, beta-hydroxy ketone	193, 139	^46^
59	17.302	371.221	Oxo-ionol- glucopyranoside	[M+H]^+^	C_19_H_30_O_7_	370.4	Megastigmane glycoside	371 [M−H]^−^	^68^
60	17.604	417.299	Sitostanol	[M+H]^+^	C_29_H_52_O	416.4	Phytosterol	399	^66,67^
61	17.776	295.242	Hydroxy octadecadienoic Acid	[M−H]^−^	C_18_H_32_O_3_	296.4	Hydroxy unsaturated fatty acid	277	^2^
62	17.818	318.212	Phytol	[M+Na]^+^	C_20_H_40_O	296.5	Acyclic diterpenoid	295, 253	^21^
63	17.822	316.1	Salisoflavan	[M−H]^−^	C_17_H_17_O_6_	317.1	Isoflavonoid	301, 286, 121, 120	^28^
64	17.905	503.299	Medicagenic acid	[M+H]^+^	C_30_H_48_O_6_	502.7	Triterpenoid	–	^12^
65	18.676	531.299	Calactin	[M−H]^−^	C_29_H_40_O_9_	532.6	Cardenolide	403, 373, 271	^64^
66	18.75	287.296	Cyanidin	[M+H]^+^	C_15_H_11_O_6_+	287.2	Anthocyanidin	259, 241, 231, 213, 150, 137	^26^
67	19.43	353.234	Monolinolenin	[M+H]^+^	C_21_H_36_O_4_	352.5	Monoacyl glycerol	261, 243	^56^
68	20.049	456.299	Oleanolic acid	[M+H]^+^	C_30_H_48_O_3_	456.7	Triterpenoid	439, 411, 393	^40^
69	20.736	309.203	Eicosenoic acid	[M−H]^−^	C_20_H_28_O_2_	310.5	Long chain saturated fatty acid	–	^58^
70	21.138	269.223	Apigenin	[M−H]^−^	C_15_H_10_O_5_	270.2	Flavonoid	150, 117	^25^
71	21.847	269.202	Margaric acid	[M−H]^−^	C_17_H_34_O_2_	270.5	Long chain saturated fatty acid	–	^58^
72	22.0095	279.288	Linoleic acid	[M−H]^−^	C_18_H_32_O_2_	280.4	Long chain unsaturated fatty acid	–	^2^
73	22.445	163.123	Umbelliferone	[M+H]^+^	C_9_H_6_O_3_	162.1	Coumarin derivative, hydroxy coumarin	119, 107, 91, 35	^37^
74	22.579	315.224	Isorhamentin	[M−H]^−^	C_16_H_12_O_7_	316.3	Flavonoid	300, 256	^23^
75	22.729	299.299	kaempferol-methyl ether	[M−H]^−^	C_16_H_12_O_6_	300.3	Flavonoid	284, 257, 151	^23^
76	24.455	339.2	Behenic acid	[M−H]^−^	C_22_H_44_O_2_	340.6	Long chain saturated fatty acid	342, 340	^57^
77	24.471	257.199	Pinocembrin chalcone	[M+H]^+^	C_15_H_12_O_4_	256.3	Chalcone	176, 144	^13^
78	26.498	367.2	Lignoceric acid	[M−H]^−^	C_24_H_48_O_2_	368.6	Long chain saturated fatty acid	–	^58^
79	26.68	395.299	Hexacosanoic acid	[M−H]^−^	C_26_H_52_O_2_	396.7	Long chain saturated fatty acid	–	^58^
80	27.419	423.299	Octacosanoic acid	[M−H]^−^	C_28_H_56_O_2_	424.7	Long chain saturated fatty acid	–	^58^

The identified compounds are numbered according to the experimental elution order. Fragmentation patterns of the principle components are described in the supplementary materials form of schematic diagrams. The peak intensity of each identified metabolite in each *Salsola* extract was mentioned in Table S1, calculated as (expressed as mg Equivalents/g dry extract). Furthermore, of the eighty identified metabolites, sixteen and twenty-nine compounds were identified in *S. tetrandra* shoots and roots, respectively. Also, twenty-seven and twenty- four phytochemicals of the total eighty metabolites were investigated in the shoots and roots of *S. vermiculata*, accordingly, whereas seventeen and thirty-nine secondary metabolites were known in the shoots and roots of *S. tetragona*.

#### Flavonoids

Flavonoids, being a commonly found secondary metabolite class in the plant kingdom, have the highest occurrence in our tested *Salsola* samples. Fourteen flavonoids were identified in our samples. They are a product of condensation between hydroxycinnamic acids and malonyl residues, comprising the structure of the C6-C3-C6 scaffold. Based on the oxidation state of ring C, they are classified, and the retro-Diels Alder (RDA) reaction is the main fragmentation pathway^18^. Compound **24** exhibited an intense peak at m/z 609 [M−H]^−^ and daughter ions 447 [M−H-162]^−^, 301 [M−H-162-146]^−^, 151 due to C-C bond cleavage at ring C^19^. Therefore, it was elucidated as hesperidin. Similarly, compound **28** was tentatively annotated as diosmin. It displayed a base peak at m/z 609 [M+H]^+^, in addition to 447 [M+H-162]^+^, 301 [M+H-162-146]^+^ for diosmetin and 191 for 7- etoxycoumarin^20^. Compounds **10** and **17** were annotated as dirhamnosyl quercetin and glucosyl rhamnosyl quercetin, respectively. They showed deprotonated quasi-molecular ions of *m/z* 593 and 609, respectively. In addition to the daughter ions [M−H-146]^−^, [M−H- 146–146]^−^ corresponding to the loss of one and two rhamnose units, respectively, [M−H-162]^−^ corresponding to the loss of a glucose unit and [M−H-162-146]^−^ relevant to the loss of glucose and rhamnose units, quercetin aglycone ions were spotted in the spectra of the aforementioned two compounds, **10** and **17**^21^. Compound **38** was elucidated as catechin due to the presence of daughter ions at 291 [M+H]^+^, 273 [M+H-H_2_O]^+^ and 139 [M+H-152]^+^ due to 1,2 cleavage of ring C^22^. Compounds **45** and **77** were elucidated as kaempferol and kaempferol methyl ether. They exhibited significant peaks of deprotonated ions [M−H]^−^ at *m/z* 285 and 299, respectively, alongside the RDA ions 257,151 and [M−H-CH_3_]^−^^23^. Compound **76** was tentatively annotated as isorhamnetin as it displayed a base peak at *m/z* 315 [M−H]^−^ and [M−H-CH3]^−^ to produce rhamnetin ion^23^. Compound **50** was elucidated as naringenin, which displayed a base peak at *m/z* 273 [M+H]^+^ and RDA ion 153^24^. Similarly, Compound **72** was identified as apigenin, as it exhibited a significant ion at *m/z* 269 [M−H]^−^, then it was subjected to 1,2 cleavage of ring C to produce RDA ion at *m/z* 117^25^. Compound **68** was annotated as cyanidin as it exhibited daughter ion peaks [M-CO]^+^, [M−H_2_O-CO]^+^, [M-CO-CO]^+^, [M−H_2_O-CO-CO]^+^ and an RDA fragments at *m/z* 259, 241, 231, 213, 150, and 137, respectively^26^. Furthermore, the mass spectrum of compound **79** was noticed to ensure the presence of daughter ions *m/z* 176 and 144, corresponding to the formation of RDA ions of chalcones, respectively. Hence, it was known as pinocembrin chalcone^13,27^.

Compound **64** displayed daughter peaks at *m/z* 316, 301, 286, 121, and 120, corresponding to [M−H]^−^, [M−H-CH_3_]^−^, [M−H-CH_3_-CH_3_]^−^, [M−H-C_10_H_11_O_4_]^−^ and [M−H-C_10_H_12_O_4_]^−^, respectively. In the same manner, as described by^28^. Compound **42**, which was identified as tri methoxy-dimethoxy isoflavone, exhibited characteristic peaks at m/z 329, 314, 299, 165, and 164 relevant to fragment ions [M−H]^−^, [M−H-CH_3_]^−^, [M−H-CH_3_-CH_3_]^−^ and RDA ions, respectively^29^.

#### Coumarins

Coumarins are a group of polyphenolic compounds characterized by the presence of a benzo-γ-pyrone ring (benzo chromone)^30^. Seven coumarins were identified in the three *Salsola* species. Coumarinolignanoids undergo fragmentation^31^. Hence, compound **6** was assigned as cleomiscon B due to the presence of daughter ions at *m/z* 367, 207, 179, 136, and 123^13^. Compound **33** was annotated as daphnoretin as it displayed a base peak at *m/z* 353 corresponding to [M+H]^+^ and daughter peaks of *m/z* 336 and 191 due to loss of the CH_3_ group and de-dimerization, respectively^32^.

Compound **37** was annotated as furanocoumarin; known as bergaptol, as it formed a protonated ion peak at *m/z* 203 and then the elimination of small molecules such as CO and 2 CO was observed^13,33^. Similarly, Compound **41**, which was elucidated as fraxetin, exhibited CH_3_ and CO molecule loss, meanwhile, Compound **47**, which was known as isofraxidin, exhibited only CH_3_ group loss^34,35^. On the other side, compound **49** base peak at *m/z* 353 exhibited a loss of 162 Da, corresponding to a hexose unit loss, followed by demethylation and loss of CO molecule; hence, it was annotated as scopoletin glycoside^36^. Compound **75** exhibited an intense peak at *m/z* 163. This peak was fragmented by eliminating CO_2_ and CO and C_2_O_2_ (−56 Da) molecules; therefore, it was annotated as umbelliferone^37^.

#### Triterpenoids

Seven triterpenoids were annotated in the sample extracts. Compound **58** displayed a peak of *m/z* 425 corresponding to [M−H]^−^ and other peaks of 407, 389, and 363 corresponding to [M−H-H_2_O]^−^, [M−H-2H_2_O]^−^ and [M−H-CH_2_CHOH]^−^, respectively. So, it was tentatively annotated as lupeol^38^.

Compounds **27**, **48**, **70**, and **52** were elucidated as glucopyranosyl oleanolic acid, olean-en-diol, oleanolic acid and salolic acid, respectively, as they displayed intense peaks at *m/z* 619, 443, 457, and 489, respectively corresponding to the protonated ions of each one. MS/MS fragmentation revealed daughter ions due to the loss of one glucose unit (−162 Da), followed by the loss of a water molecule and formic acid (−46 Da)^39,40^. Compound **36** was tentatively identified as salsolin A. It exhibited a base peak of *m/z* 504 corresponding to [M+H]^+^, followed by the loss of one carboxyl group (−46 Da) with the characteristic retro-Diels-Alder fragments at *m/z* 224 and 280, corresponding to the two hydroxyl groups in ring A and B and two hydroxyl groups and a carboxyl group in ring C and D or E^41^. Compound **65** exhibited an intense peak at *m/z* 503, which conforms to a molecular formula of C_30_H_47_O_2_. Therefore, it was tentatively identified as mediagenic acid^12^.

#### Phenolics and phenolic acids

Phenolic acids represent a major class of secondary metabolites. They are distributed in our *Salsola* samples as seven phenolic compounds and five phenolic acids. Compound **40** exhibited an intense peak at *m/z* 359; its MS/MS fragmentation revealed the formation of 3-(2,3-dihydroxy-phenyl) lactic acid (*m/z* 197), caffeic acid (*m/z*179) and their dehydrated form (*m/z* 161). Therefore, it was tentatively identified as rosmarinic acid^42^. Compounds **25**, **46**, **54**, and **57** were tentatively identified as tachioside, dihydroxy phenyl-ethyl-β-D-glucopyranoside, cunataside C and canthoside D as they exhibited precursor ions at *m/z* 301,315, 551,433, respectively, followed by loss of glucose unit [M−H-162]^−^^13,43,44^. Compound **34** showed an intense peak at *m/z* 164 [M+H]^+^ and a daughter peak at *m/z* 146; hence, it was elucidated as p-coumaramide^45^. Compound **59** was identified as gingerol as it displayed a precursor ion peak at *m/z* 293 [M−H]^−^ and daughter ions at *m/z* 193 and 139 due to the cleavage of C4-C5 bond^46^.

Each phenolic acid molecule is characterized by the presence of hydroxylated aromatic rings linked to a carboxyl group. They occur in the form of C6-C1 and C6-C3. Compound **8** displayed a deprotonated ion (*m/z* 179) that was fragmented to form the thermodynamically most favorable pathway of CO_2_ loss (−44 Da) and a consecutive loss of CO molecules (−28 Da). This peak ion, in another pathway, showed H_2_O molecule loss (*m/z* 161) through triple bond formation (C2 or C6) by remote hydrogen re-arrangement, so it was tentatively annotated as caffeic acid^47^. Similarly, compound **7** was identified as orsellic acid, as its deprotonated ion exhibited CO_2_ loss, followed by an H_2_O molecule in one pathway and an H_2_O molecule in the other one^48^. In addition to that, compound **4**, which was annotated as hypogallic acid, showed an intense deprotonated peak at *m/z* 153, followed by a loss of CO_2_ molecules^49^ and compound **13** showed a deprotonated ion at *m/z* 183, followed by the loss of CH_3_, CH_3_ and CO and CH_3_ and CO_2_ molecules, so it was known as O-methyl gallic acid^50^. Compound **9** was annotated as anisic acid as its protonated ion exhibited a loss of CO_2_ molecules besides the formation of protonated carbon dioxide (HO + C ≡ O, *m/z* 45)^51^.

#### Nitrogenous compounds

Eleven nitrogenous compounds were identified in *Salsola* extracts. Compounds **16**, **19**, **21** and **22** were characterized as tyramine derivatives, as their MS/MS fragmentation displayed fragments corresponding to tyramine moiety (m/z 136) and loss of caffeoyl moiety [M−H-166]^−^ for N-caffeoyl tyramine and feruloyl moiety [M−H-177]^−^ for N-feruloyl tyramine and loss of feruloyl and methoxyl groups [M−H-177]^−^ and [tyramine moiety-31]^−^ for N-feruloyl-3′′′-methoxy tyramine and loss of hydroxy feruloyl moiety [M−H-193]^−^for hydroxy moupinamide^2^. Compound **14** was characterized as feruloyl octopamine as it exhibited a protonated base peak and daughter ions relevant to the loss of the feruloyl moiety and the octopamine moiety, which in turn showed the loss of a water molecule to produce the tyramine moiety. Similarly, compound **12**, which was identified as N-(4’-methoxcinnamoyl)-norepinephrine, showed a precursor ion at m/z 328 [M−H]^−^ and daughter ions at m/z 153 due to the loss of methoxy cinnamoyl and m/z 107 corresponding to norepinephrine^13^. Compounds **5** and **29** were elucidated as pericamylinone A and salasmine, respectively. Their MS/MS fragmentation spectra exhibited the characteristic RDA fragments at *m/z* 192, 178, and 149^13^. Compound **39** exhibited a loss of 132 Da, corresponding to a ribose sugar unit. Therefore, it was elucidated as uridine, as described by^52^. Compound **3** protonated precursor ion exhibited a characteristic acetyl group loss to produce a daughter ion fragment at *m/z* 58. Therefore, it was elucidated as betaine^53^. Compound **18** was tentatively annotated as hernandine because of the characteristic fragmentation pattern of protonated aporphine alkaloids [M+H]+, [M+H-CH_3_]+, [M+H-CH_3_-CH3OH] and [M+H-CH_3_OH-CO]^54^.

#### Fatty acids

*Salsola* species are rich in fatty acids and eleven fatty acids were identified in our samples. Compound **20** was elucidated as a dicarboxylic acid metabolite (Azelaic acid), as it exhibited a deprotonated ion fragment at *m/z* 187, with a sequential loss of H_2_O, CO_2_ and H_2_O + CO_2_ molecules^55^. Compound **62**, which was known as hydroxy octadecanoic acid, showed an intense peak at *m/z* 295 corresponding to [M−H]^−^, accompanied by a loss of water molecule (−18 Da), which was an indicator of the presence of one hydroxyl group, yielding a daughter peak at *m/z* 277 corresponding to octadecadienoic acid. In the same way, compound **43** MS/MS fragmentation spectrum revealed three consecutive water molecules loss. Hence, it was elucidated as trihydroxydecosan-ienoic acid^2^. Compound **69** displayed a precursor ion peak at *m/z* 353 [M+H]^+^. Further fragmentation gave rise to daughter peaks at *m/z* 261 as a result of a glycerol moiety loss and 243 due to a water molecule loss. Therefore, this compound was annotated as 9,12,15-octadecatrienoic acid, 2,3-hydroxypropyl ester (1-monolinolenin)^56^. The base peak is the most sensitive peak for free fatty acid detection. Hence, compounds **71**, **73**, **74**, **78**, **80**, **81** and **82** were annotated as ecosenoic acid, margaric, linoleic, behenic, lignoceric, hexacosanoic and octacosanoic acids, respectively^57,58^.

#### Saponins

Four saponins were annotated in *Salsola* extracts. Compounds **30**, **31** and **35** were tentatively elucidated as salsoloside D, salsoloside E and salsoloside C, respectively. They exhibited base peaks at *m/z* 941, 1087 and 925, respectively. MS/MS fragmentation spectrum of compound **30** revealed a hedragenin aglycone ion moiety (*m/z* 471) alongside other ions of m/z [M−H-162]^−^, corresponding to the loss of a hexose unit, [M−H-162-132]^−^, corresponding to the loss of a pentose unit and [M−H-162-132-176]^−^, corresponding to the loss of one glucuronic acid molecule^6,59^. Compounds **31** and **35** MS/MS fragmentation revealed an oleanolic acid ion (m/z 455), [M−H-162]^−^, [M−H-162-162]^−^, [M−H-162-162-132]^−^ and [M−H-162-162-132-176]^−^ as compound **30**^6^. Compound **32** showed a base peak at m/z 681 [M−H]^−^ and daughter ions at m/z 519 [M−H-162]^−^ and 471[M−H-162-HCHO-H_2_O]^−^; therefore, it was elucidated as pentahydroxy-oleanen-oic acid glucopyranoside^13^.

#### Sugars

Compound **1** showed an intense peak at *m/z* 365, which corresponds to the formation of the [M+Na]^+^ adduct of trehalose. Further fragmentation of these adducts yielded another peak at *m/z* 203, which corresponds to the formation of the [M+Na]^+^ adduct of glucose^60^. On the other hand, Compound **2** displayed a precursor ion peak at 193 corresponding to [M−H]^−^ and daughter ions corresponding to a non-reducing end and a reducing end, respectively. So, it was tentatively annotated as galacturonic acid^61^.

#### Cardenolides

Caredenolides are a group of cardiac active metabolites. They are characterized by the presence of a steroidal nucleus, a five-membered lactone ring and an oligosaccharide moiety attached to position 3^62^. Cardenolides displayed very similar MS/MS spectra. Compounds **26** and **67** were elucidated as salsoteragonin and calactin, respectively. That was observed through the loss of sugar units, H_2_O and CO molecules^13,63,64^.

#### Miscellaneous compounds

Compound **56** was elucidated as hydroxy pseudoguaien olide didehydro ketone as it showed an intense peak at *m/z* 263 corresponding to [M+H]^+^ and daughter peaks [M+H-H_2_O]^+^, [M+H-H_2_O-CH3]^+^ and [M+H-H_2_O-CH_3_-CO]^+^^65^. Compound **61** was annotated as sitostanol. Phytosterol displayed similar mass spectra, including the loss of water molecules and the cleavage of C17 and C20. Therefore, the obtained ions 417 [M+H]^+^ and 399 [M+H-H_2_O]^+^ confirmed the presence of sitostanol^66,67^. Compounds **53**, **55** and **60** were tentatively annotated as blumenyl glucopyranoside, staphylinoside D and oxo-ionol glucopyranoside, respectively, with the reference to literature, as they exhibited precursor ion peaks at *m/z* 387, 385 and 371, respectively^68^. Compound **63** was tentatively annotated as phytol, displaying a prominent peak at *m/z* 318 [M+Na]^+^. It was.

confirmed from data on Mass Bank and NIST database alongside the literature^21^.

### Comprehensive metabolomic study of the shoot and root samples of the three *Salsola* species

**Fig. 1:**
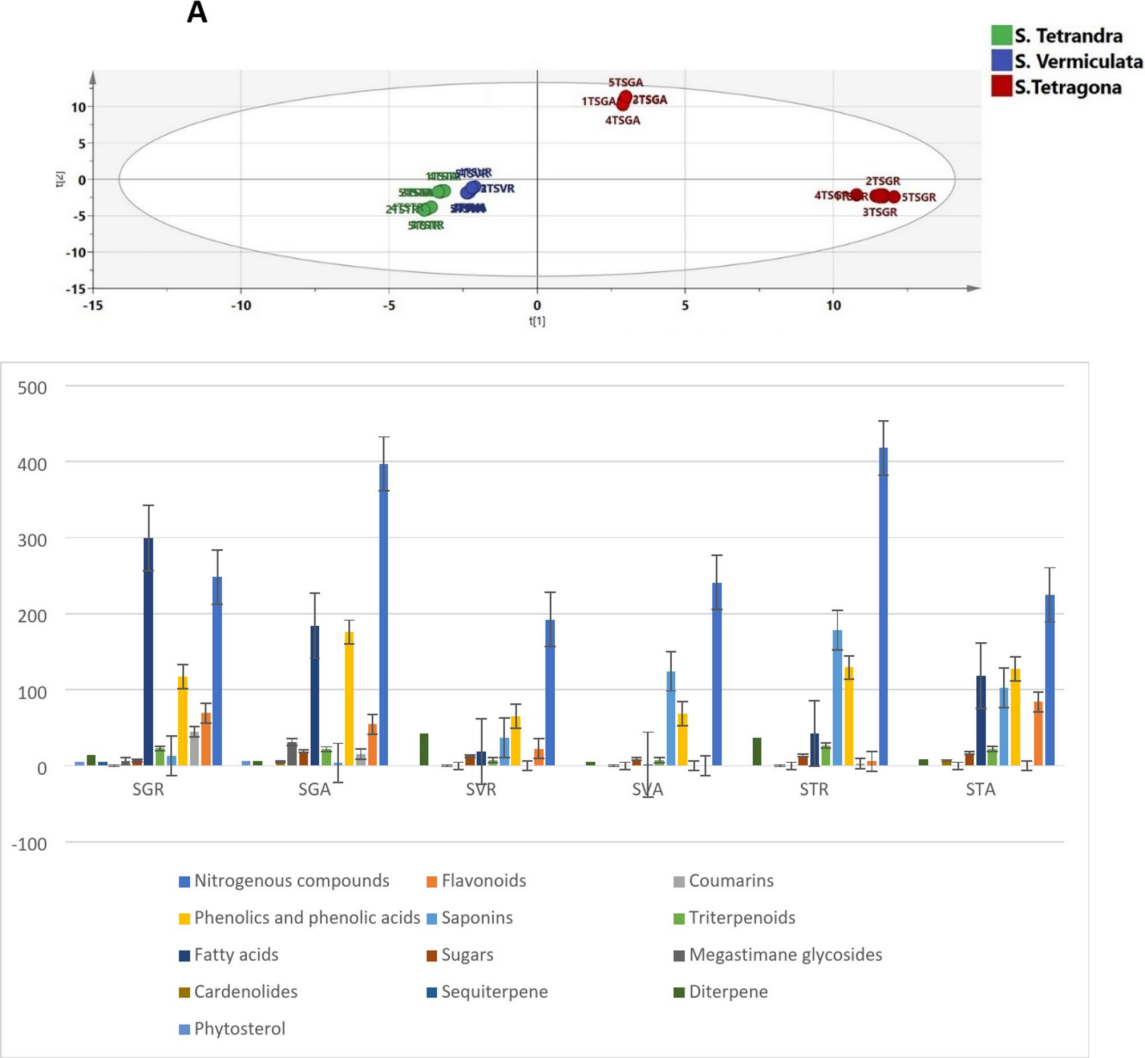
(**A**) Principle component analysis (PCA) score scatter plot of *Salsola* extracts, (**B**) Relative quantitation of the chemical classes in the tested *Salsola* samples in mg equivalent (Eq.)/g dry weight.

A metabolomics-based approach linked to multivariate analysis was implemented to explore the clustering pattern of the three *Salsola* species. Unsupervised recognition analysis was performed by constructing the principal component analysis (PCA) score scatter plot model. PC1 and PC2 represented 69.75% of the total variation. Model validation was ensured as R^2^ (goodness of fit) and Q^2^ (goodness of prediction) were valued at 1 and 0.999, respectively, proving the model’s reliability for fitting and predicting future samples. As depicted in Fig. 1A, *Salsola* samples were segregated into three clusters, revealing the variation in their chemical matrices. PC2 separates *S. tetragona* from *S. tetrandra* and *S. vermiculata*. *S. tetragona* is the only species showing separation between its organs in the PCA, although the other two species were clustered together in the form of a single holistic cluster combining both shoot and root extracts of both species. The samples of *S. tetragona* roots were clustered along the positive side of PC1 and the negative side of PC2, meanwhile, the shoots of the same species were clustered along the positive sides of both PC1 and PC2. *S. tetrandra* and *S. vermiculata* shoot and root samples were clustered along the negative sides of both PC1 and PC2. Principal components 1 and 2 were used to classify the extracts according to the quartile where they are clustered, hence the clustering quartiles were referred to as the positive and negative side in relation to each PC. The positive side of both was the upper right quartile and the negative side of both was the lower left quartile.

**Fig. 2:**
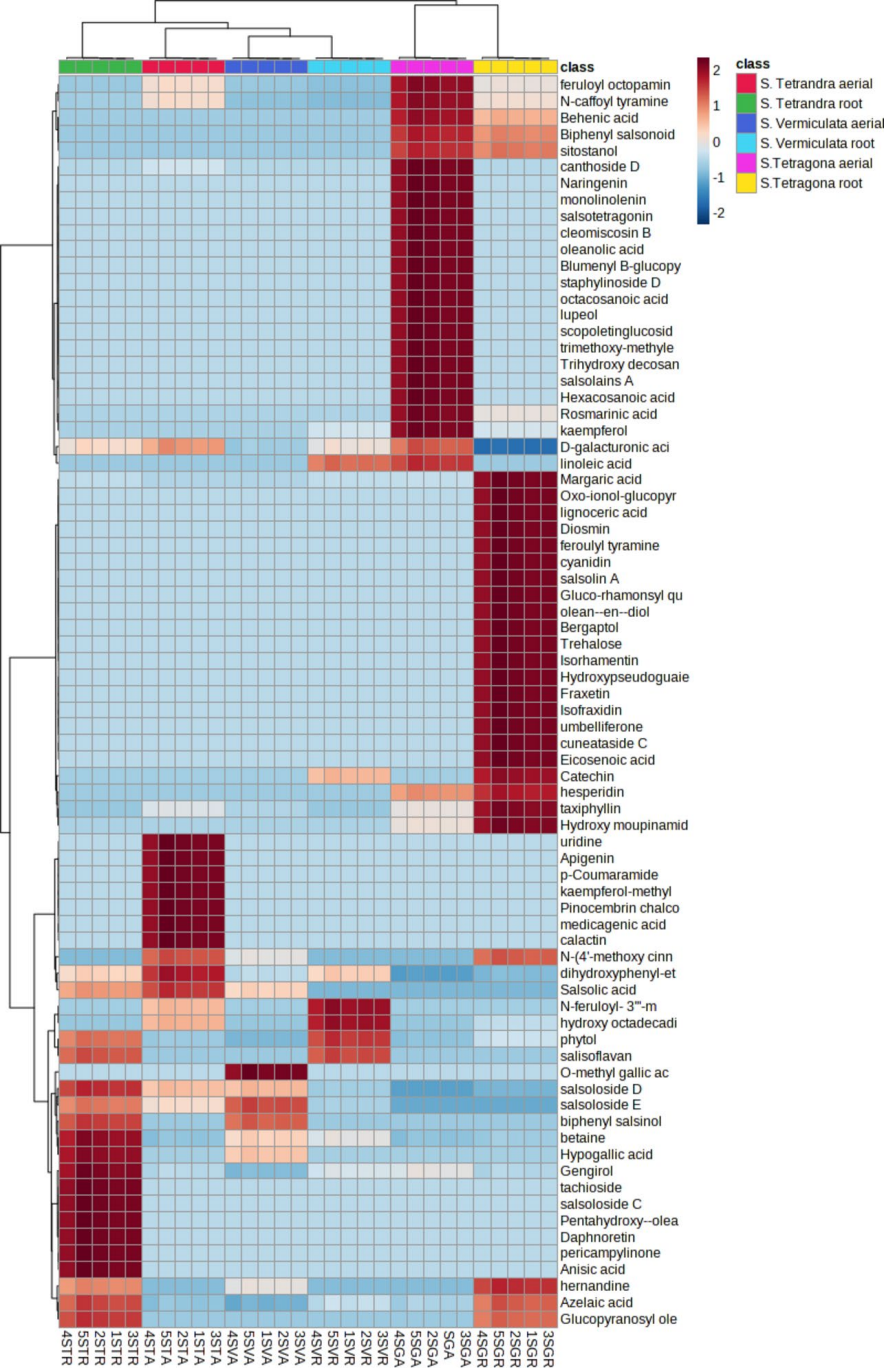
Hierarchical clustering heat map (HCA) analysis of* Salsola* species.

Semi-quantitative data on the identified metabolites (Table S2) was used to construct a hierarchical cluster analysis heat map (HCA). As depicted in Fig. 1B, the highest amount of nitrogenous compounds was detected in the root sample of *S. tetrandra* and the shoot sample of *S. tetragona*, meanwhile, phenolics and phenolic acids were mostly detected in the shoot sample of *S. tetragona* and the samples of both organs of *S. tetrandra*. The shoot sample of *S. tetrandra* overwhelmed the vast quantity of flavonoids. It is clear that the proportion of most classes was markedly different between the shoot and root samples of *S. tetragona*, particularly fatty acids, nitrogenous compounds and phenolics. The root sample of *S. tetragona* possessed double the amounts of fatty acids compared to the shoots. On the other hand, *S. tetragona* shoots contained roughly double the amounts of phenolics and nitrogenous compounds in comparison to the same species’ root. It is worth mentioning that megastigmane glycosides were exclusively detected in the shoot and root samples of *S. tetragona*. Back to the literature, no previous studies could present a comprehensive profiling of that species, giving us the credit of providing the first attempt for investigating *S. tetragona*. *S. tetrandra* and *S. vermiculata* shared a common chemical profile, recording comparable amounts of triterpenoids and phenolics. A previous study utilized the LC-MS/MS technique to unravel the chemical complexity of *S. tetrandra* and *S. vermiculata* and recommended further investigation of their chemical profile^2^. The study in hand, was able to deeply compare between the two species and ensured the chemical similarities between them. Based on the aforementioned discussion, the marked variation in the chemical profile between the species of *S. tetragona* and the noticeable similarities among *S. tetrandra* and *S. vermiculata* samples were the reasons behind the clustering pattern. The heat map (Fig. 2) represented the relative concentration of the identified metabolites in each extract. Each standard of certain chemical class was used to calculate the relative concentration of the compounds of the same chemical class by dividing the peak area of the compound over the peak area of the standard, then utilizing the slope and intercept of each standard, the concentration of each metabolite of the same chemical class was calculated as mg/equivalent, then the value was divided by the amount of the total extract, which is 0.02 g (the amount used in the UHPLC study from each extract), and the value would be mg equivalent (Eq.)/g dry weight dry extract.

As depicted in Fig. 2, the species effect had the upper hand in the clustering pattern of the investigated *Salsola* species, holding a great variation in the chemical profile of *S. tetragona* and some similarities in the profiles of *S. tetrandra* and *S. tetragona*. The organ effect was observed in the sub-clustering pattern of each species.

Eight compounds of the eighty identified metabolites were common in both organs of the three tested species; two nitrogenous compounds, betaine and feruloyl octopamine, had the highest occurrence in *S. tetrandra* roots and *S. tetragona* shoots, respectively. The saponin salsolside D, dicarboxylic azelaic acid and the phenolic compound gingerol possessed the highest prevalence in *S. tetrandra* roots. The acyclic diterpene phytol was mostly detected in *S. vermiculata* roots. The phenolic compound dihydroxyphenyl-ethyl glucopyranoside was highly concentrated in *S. tetrandra* shoots. Moreover, D-galacturonic acid was common in all samples but was accumulated in comparable concentrations in both *S. tetragona* and *S. tetrandra* shoots.

Also, studying Fig. 2 revealed that N-caffeoyl tyramine, hydroxy octadecadienoic acid and dirhamnosyl quercetin were the major compounds in *S. tetrandra* shoot samples. On the other hand, salsoloside E, glucopyranosyl oleanolic acid and hypogallic acid were the main metabolites in *S. tetrandra* root samples. Whereas salsoloside E, hypogallic acid and O-methyl gallic acid were the main chemical components in *S. vermiculata* shoot samples and hydroxy octadecadienoic acid, catechin and salsoloside E were the major constituents in *S. vermiculata* root samples. On the contrary, hexacosanoic acid, canthoside D, N-caffeoyl tyramine and behenic acid were the major secondary metabolites in *S. tetragona* shoot samples, meanwhile, margaric acid, eicosenoic acid, taxiphyllin and hydroxy octadecadienoic were the major chemical compounds in *S. tetragona* root sample.

**Fig. 3:**
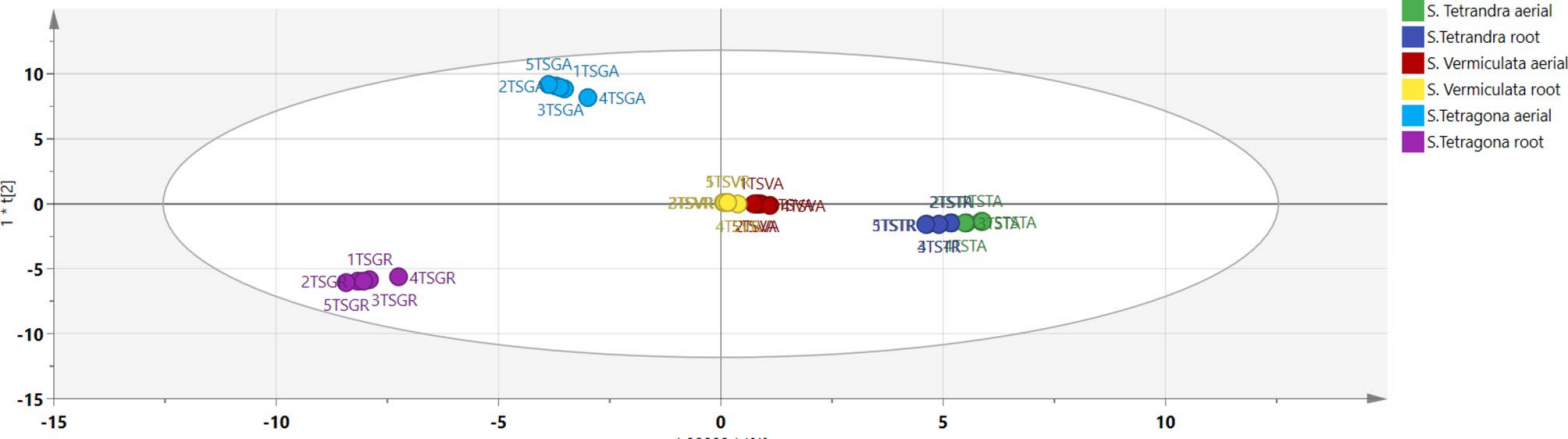
OPLS DA score scatter plot models of *Salsola* species.

Investigating the chemical matrices of each species’ extract, orthogonal projection to latent structure discriminatory analysis (OPLS DA) scores scatter plot models of total extracts to assign any in-between or within-class discrimination. The species was used as the main class identifier for constructing a validated discriminatory model. The validation of the model was assured with R^2^X, R^2^Y and Q^2^ values of 1,1,0.999. As observed in Fig. 3, the shoot and root extracts of *S. tetrandra* were clustered together and both organs of *S. vermiculata* were clustered together. Both species exhibited two separated clusters on the positive side of LV1 and the negative side of LV2, controversy, the roots extract of S. tetragona was completely separated from the shoots, as the shoot extract of S.tetragona was clustered along on the positive side of LV2 and the negative side of LV1 and the root extract of the same species was clustered along the negative sides of both LV1 and LV2. To sum it up, OPLS DA confirmed that the species effect has been profoundly able to discriminate between the three species, recording a great variation in the chemistry between the shoots and roots of *S. tetragona* and some similarities between *S. tetrandra* and *S. vermiculata*.

The coefficient plots find a proportional correlation of the amount of any compound in the extract; therefore, as the length of the bar corresponding to a certain metabolite was the highest, that meant the high abundance of that compound in the extract.

As depicted in Fig. S2, OPLS DA coefficient plots revealed that salsoloside D, salsoloside E, salsolic acid and gingerol were positively correlated for the discrimination of *S. vermiculata* shoots; meanwile, betaine, catechin, salisoflvan, margaric acid, linoleic acid and N-feruloyl-3′′′-methoxy tyramine were positively attributed to the species roots. Moreover, D-galacturonic acid, cleomiscosin B, N-caffeoyl tyramine, feruloyl octopamine and monolinolenin were discriminatory metabolites of *S. tetragona* shoots, while trehalose, azelaic acid, dihydroxy phenyl-ethyl glucopyranoside, eicosenoic acid, cuneataside D, salsoloside D, Oxo-ionol glucopyranoside, hydroxy octadecadienoic acid and margaric acid were strongly correlated to that species roots. In addition to that, taxiphyllin, hydroxy octadecadienoic acid, salsoloside D, salsoloside E and pinocembrin chalcone were positively correlated to *S. tetrandra* shoots; betaine, hypogallic acid, anisic acid, pericampylinone A, hernandine, tachioside, glucopyranosyl oleanolic acid, pentahydroxy oleanen-oic acid glucopyranoside, salsoloside C and azelaic acid were strongly correlated to *S. tetrandra* roots.

### Anti-inflammatory activity of *Salsola* extracts

**Fig. 4:**
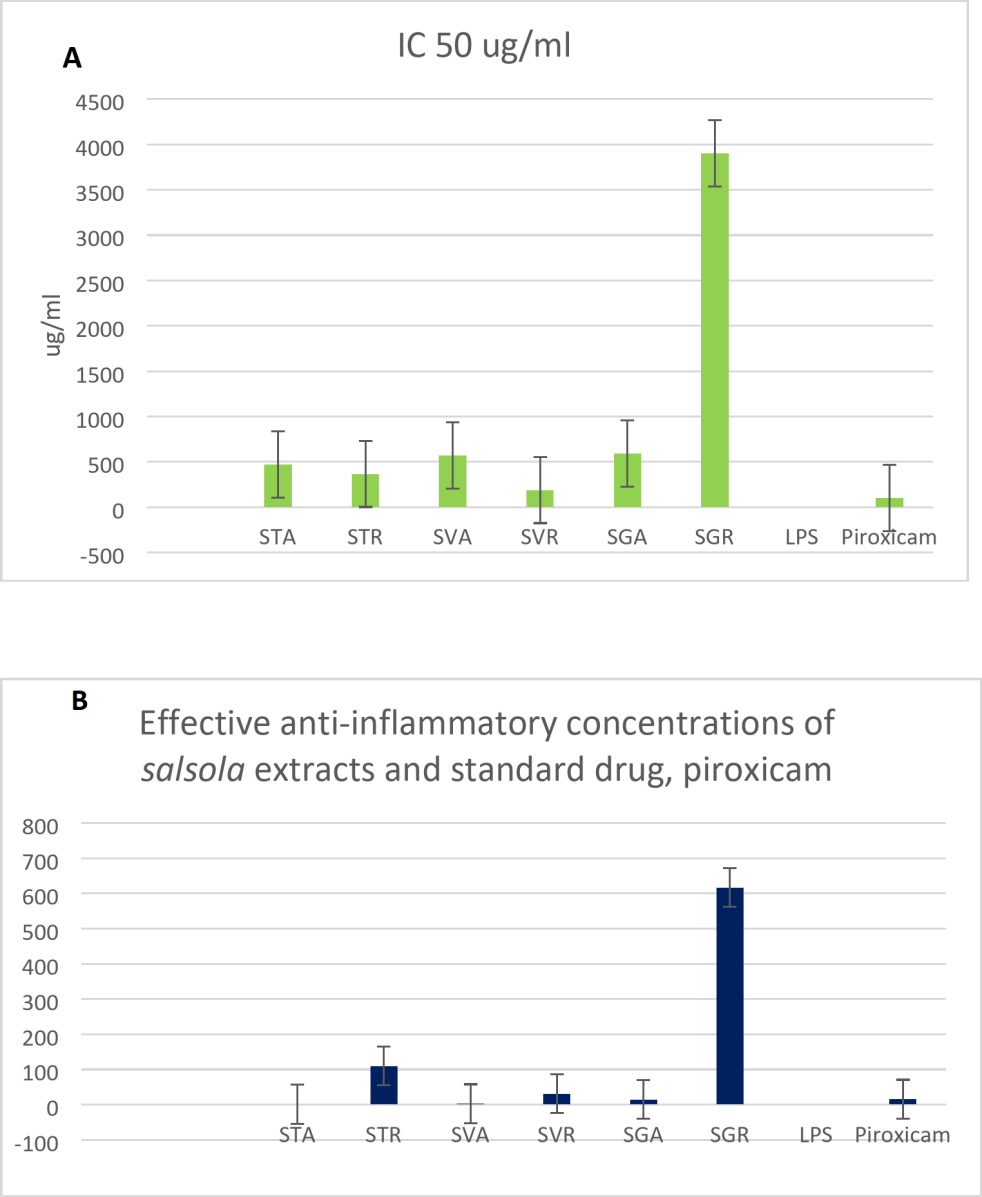
(**A**) IC50 of all Salsola extracts and standard drug piroxicam, (**B**) Effective anti-inflammatory concentrations (EAIC) of Salsola extracts and standard drug, piroxicam.

**Fig. 5:**
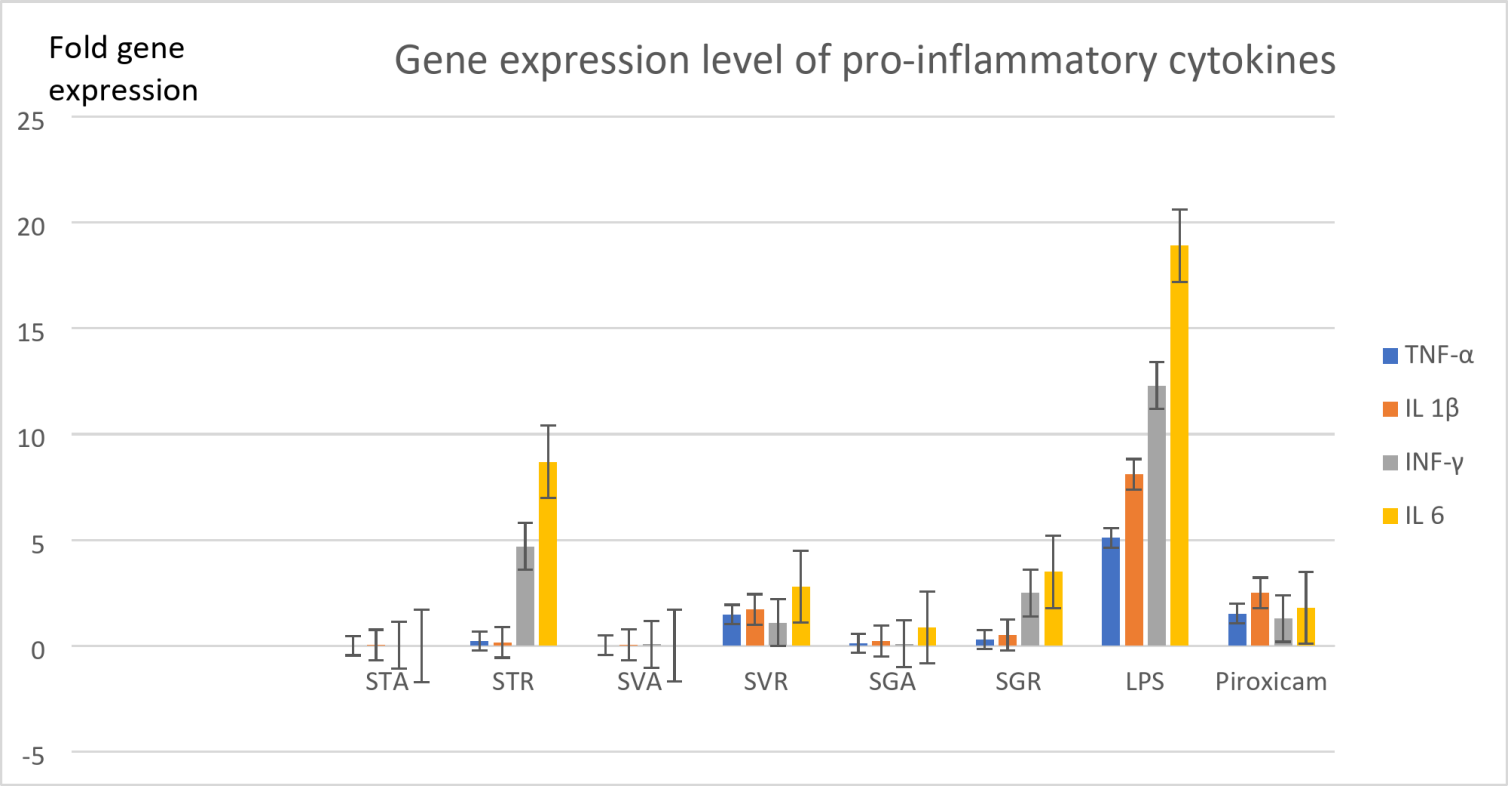
The fold gene expression of four pro-inflammatory mediators, TNF-α, IL 1β, IL 6 and INF-γ in the presence of *Salsola* extracts and piroxicam.

Determining a safe dose of each extract is a crucial step in the biological scanning process to exclude any toxicological interference. Hence, the MTT assay was carried out to assign the dose producing 100% viability of the tested WBCs (IC_50_), where high values determine high safety. It was observed in Fig. 4A, that IC_50_s of all extracts were relatively higher than the standard anti-inflammatory drug piroxicam, which indicates a high safety margin. The extract of *Salsola tetragona* roots showed an 8-fold higher IC_50_ than the piroxicam. Moreover, all *Salosla* extracts exhibited higher safety margins than the standard chemical drug. The results were in agreement with previous publications on the safety profile of *Salsola*^69,70^. To date, the study in hand has provided safety evidence for the three investigated species for the first time. 1/10 of the IC_50_ dose of each fraction was used to evaluate its anti-inflammatory activity by calculating the EAIC. EAIC is defined as the extract concentration required to bring the LPS-stimulated WBCs to the normal proliferation condition, considering that potent activity is reflected by low EAIC values. As depicted in Fig. 4B, the shoots of the three species demonstrated higher activities than the roots and piroxicam, recording 1.02, 2.67 and 14.6 μg/ml for *S. tetrandra*, *S. vermiculata* and *S. tetragona*, respectively, while the standard recorded 15.43 μg/ml and the root extracts recorded results over 100 μg/ml, revealing the potent anti-inflammatory activity of *S. tetrandra*, *S. vermiculata* and *S. tetragona* shoot extracts as they were able to bring the LPS-stimulated WBCs to normal proliferation in very low concentrations compared to that recored by the standard drug, piroxicam and the weak activity of the roots, which were used in concentrations more than six folds than the reference to normalize the proliferation of stimulated cells. Tracking the chemical profiles of these active extracts revealed their overwhelming richness in polyhydroxylated metabolites, especially flavonoids and saponin glycosides. Four pro-inflammatory biomarkers TNF-α, IL1*β*, IL6 and INF-γ, were measured in the presence of each extract and piroxicam through a mechanistic study searching for the best anti-inflammatory drug candidate. This procedure included the utilization of LPS to stimulate the cultivated WBCs and then adding the extract and the reference to screen the activity. Biochemically, LPS is a part of the cell wall of gram-negative bacteria, comprising three parts lipid: A, core protein and O-antigen. Lipid A stimulated WBCs and the aforementioned four pro-inflammatory mediators were released^15^. Regarding Fig. 5: the fold gene expression level of the four pro-inflammatory cytokines was significantly downregulated in the presence of the shoot extracts of the three species compared to roots and piroxicam, spotting out the potential anti-inflammatory activity of the shoot extracts and their ability to decrease the expression level of the four pro-inflammatory biomarkers. Among all the extracts, the shoot extract of S. tetrandra was the most biologically active extract as the fold expression of TNF-α, IL 1β, INF-γ and IL 6 was 0.01256, 0.04, 0.03 and 0.002728 fold, respectively. Furthermore, in the presence of all other extracts the expression level was higher and even in the presence of piroxicam, which recorded 1.53, 2.5, 1.3 and 1.8 fold for TNF-α, IL 1β, INF-γ and IL 6. Therefore, this ex-vivo study revealed the potential anti-inflammatory activity of *S. tetrandra* and its potent activity that exceeded the activity of piroxicam.

### Predicting the anti-inflammatory activity of *Salsola* extracts

**Fig. 6:**
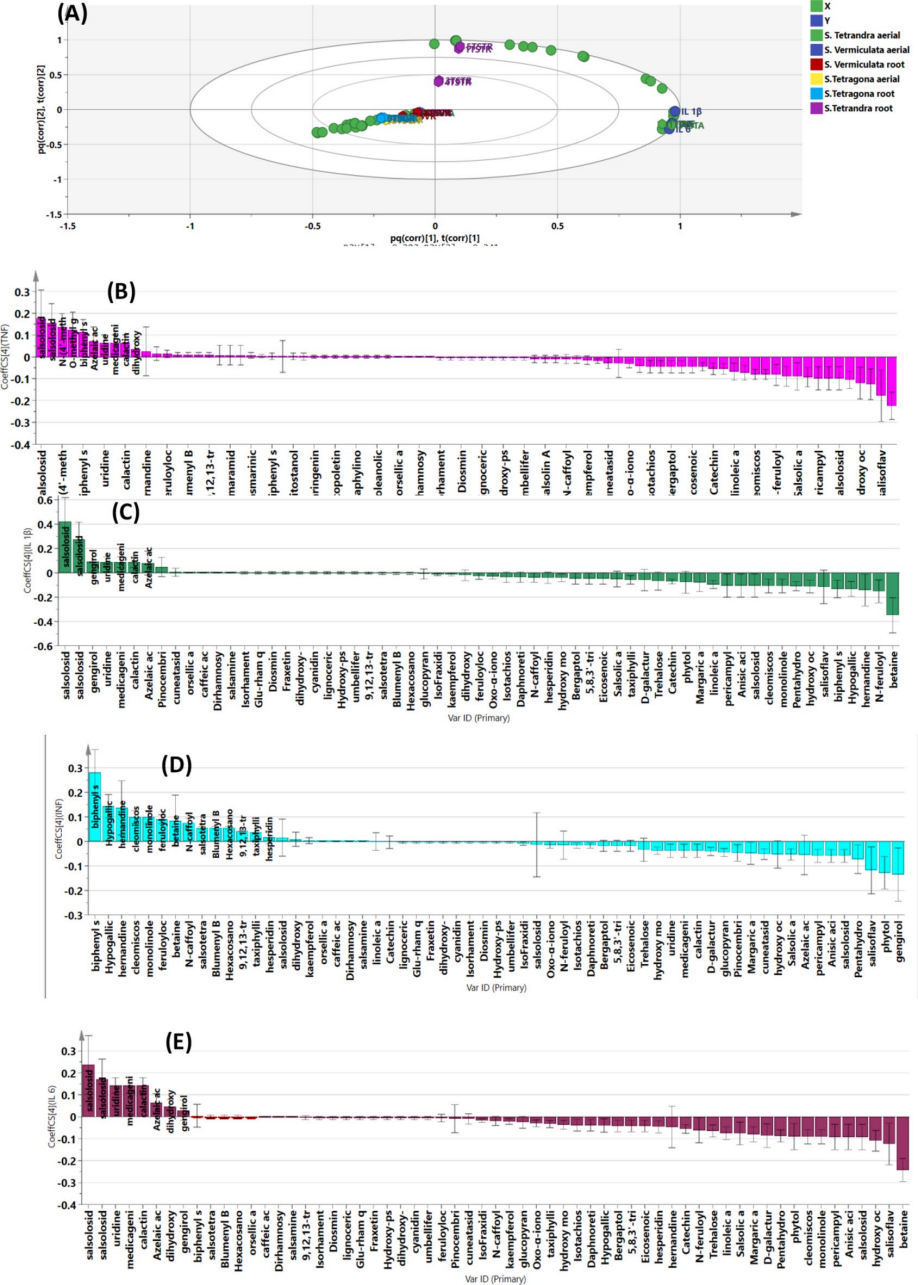
OPLS biplot of* Salsola* samples in correlation to the pro-inflammatory markers inhibition level (**A**) and correlation analysis of the identified metabolitesand TNF-α (**B**), IL 1β (**C**), INF-γ (**D**) and IL 6 (**E**).

An OPLS biplot was constructed to correlate the tested *Salsola* samples to the investigated pro-inflammatory cytokines. Figure 6 and S3 reveal the model validation for prediction. R^2^X, R^2^Y and Q^2^ were 0.958, 0.988 and 0.986, respectively. Furthermore, four permutation plots were constructed for each investigated cytokine, where R^2^ and Q^2^ of the permuted data were less than 0.6 and 0, respectively, in each model, excluding data overfitting and confirming the validity. Studying Fig. 6 revealed that the shoot extract of *Salsola tetrandra* was the most biologically active extract due to its close proximity to the investigated pro-inflammatory mediators. Also, 4-methoxy cinnamoyl norepinephrine was positively correlated to the inhibition of the gene expression levels of TNF-α, IL6 and INF-γ. Moreover, salsolosides D and E were positively correlated to TNF-α, IL1*β* and IL 6 gene expression downregulation. Biphenyl salsinol was positively correlated with inhibiting TNF-α and INF-γ gene expression levels, meanwhile, calactin and medicagenic acid were positively correlated to downregulate IL6 expression levels. In addition, 4-O-methyl gallic acid, hypogallic acid and hernandine were exclusively correlated to inhibit INF-γ gene expression level. The anti-inflammatory activities of these biomarkers were reported in the literature. These anti-inflammatory markers have previously been reported to exert an in-vitro/in-vivo anti-inflammatory effect. Regarding the nitrogenous compounds, N-4-methoxy cinnamoyl norepinephrine was proven to inhibit the release of TNFα, IL1*β*, INFγ and IL6^13^ and that chemical compound’s antioxidant activity was clearly reported by ^71^. Hernandine anti-inflammatory effect was reported by^12^. Moreover, medicagenic acid was reported to have a significant anti-inflammatory effect in *S. cyclophylla* holistic extract^12^. In addition to that, the anti-inflammatory activity of triterpene glycosides was reported to be comparable to that produced by glucocorticoids^72^. The anti-inflammatory activity of the cardenolides calactin and the phenolic compound biphenyl salsinol was statistically reported for the first time and further biological investigations regarding their possible mechanism of action are required.

## Conclusion

In our study, three *Salsola* species, namely, *S. tetrandra*, *S. tetragona* and *S. vermiculata* shoots and roots were comprehensively chemically profiled for the first time using an ultra-high performance liquid chromatography-mass-mass spectrometry (UHPLC-MS/MS) method. Eighty metabolites were tentatively identified in our investigated extracts, where flavonoids, saponins, nitrogenous compounds, phenolics and phenolic acids were detected in all *Salsola* samples, except for megasigmane glycosides, which were detected only in *S. tetragona* samples. HCA revealed that the species effect was profoundly observed in the clustering patterns of *Salsola* extracts, while the organ effect was noticed in the sub-clustering pattern of each species. OPLS DA models revealed in-between and within-class discrimination between the two organs of each species. The ex-vivo anti-inflammatory activity of the tested samples was screened for the first time, demonstrating the mechanism of action of each extract on the gene expression level. It is worth mentioning that *Salsola* extracts represented candidates for a remarkably safer anti-inflammatory drug than the chemical standard piroxicam, whereas all extracts. The shoots of the three species exerted more potent anti-inflammatory activity than the roots. Using a chemometric-based statistical analysis, it was found that the shoot extract of *S. tetrandra* was the most biologically active extract.

## Electronic supplementary material

Below is the link to the electronic supplementary material.

Supplementary Material 1

## Data Availability

The datasets used to support this study are available from the corresponding author upon request and after satisfying ethical requirements for their release.

## Abbreviations

STA: *Salsola tetrandra* shoots
STR: *Salsola tetrandra* roots
SVA: *Salsola vermiculata* shoots
SVR: *Salsola vermiculata* roots
SGA: *Salsola tetragona* shoots
SGR: *Salsola tetragona* roots
