# Supplementary material for: A comprehensive metabolomic study of three Egyptian Salsola species revealed their potential anti-inflammatory activity
Source: Sci Rep. 2025 Feb 11;15:5056. doi: 10.1038/s41598-024-80807-2 (PMC11814127; doi:10.1038/s41598-024-80807-2)
Supplement: Supplementary file 1 — Supplementary Material 1 [file 41598_2024_80807_MOESM1_ESM.pdf]

A comprehensive metabolomic study of three Egyptian Salsola species revealed their potential anti-inflammatory activity

Abdelrhman Zakaria[a], Fahima. F. Kassem[a], Doaa A. Ghareeb[b], Safa M. Shams Eldin[a,2], Dina A. Selim[a,1,*,2]

[a] *Department of Pharmacognosy, Faculty of Pharmacy, Alexandria University, Egypt*

[b] *Bio-Screening and Preclinical Trial Lab, Biochemistry Department, Faculty of Science, Alexandria University, Alexandria, Egypt*

Center of Excellence for Drug Preclinical Studies (CE-DPS), Pharmaceutical and Fermentation Industry Development Center, City of Scientific Research & Technological Applications (SRTA-city), New Borg El Arab, Alexandria, Egypt

Research Projects unit, Pharos University in Alexandria, Egypt.

*Correspondence to: Department of Pharmacognosy, Faculty of Pharmacy, Alexandria University, Alexandria 21521, Egypt.

E-mail address: Dina.seleem@alexu.edu.eg, Dinaselim2157@gmail.com (Dina A. Selim).

[1] 002-0119944042

[2] Authors contributed equally to the manuscript.

Samples and standards preparation for UPLC-MS analysis

The procedure was performed as follows: Dilution of each extract in HPLC-grade was in methanol followed by filtration through a membrane disc filter of 0.2 m pore size, then degassing by sonication, and finally, 10 µL of the sample volume was loaded at a concentration of 1 mg/ml on the reversed-phase column in the full loop mode injection. The injection process for each sample occurred in five replicates. The regression equation is y=ax+b, where y is the peak area, x is the standard concentration in mg/ml, a is the slope, and b is the intercept.

Table S1: UHPLC-MS metabolite profiling data from all samples representing the compound variables (peak areas) calculated as (expressed as mg Equivalents/ g dry extract).

Identified Compound	STA	STR	SVA	SVR	SGA	SGR
Trehalose*	0.0	0.0	0.0	0.0	0.0	3.5
D-galacturonic Acid*	16.8	13.3	8.8	12.6	19.1	3.4
Betaine**	113.2	392.4	224.6	185.3	111.8	125.9
Hypogallic acid*	0.0	23.8	10.9	0.0	0.0	0.0
Pericampylinone-A**	0.0	3.1	0.0	0.0	0.0	0.0
Cleomiscosin B***	0.0	0.0	0.0	0.0	8.9	0.0
Orsellic acid*	4.1	0.0	0.0	0.0	0.0	0.0
Caffeic acid*	4.9	0.0	0.0	0.0	0.0	0.0
Anisic acid*	0.0	3.9	0.0	0.0	0.0	0.0
Dirhamnosyl quercetin *****	33.1	0.0	0.0	0.0	0.0	0.0
Biphenyl salsinol*	0.0	3.6	3.3	0.0	0.0	0.0
N(4'methoxcinnamoyl)-norepinephrine**	6.2	0.0	2.6	0.0	0.0	6.0
O-methyl gallic acid*	0.0	0.0	9.4	0.0	0.0	0.0
Feruloyl octopamine**	63.7	10.6	9.6	4.0	176.1	54.1
Taxiphyllin*	13.3	0.0	5.3	0.0	16.0	59.2
Hydroxy moupinamide**	0.0	0.0	0.0	0.0	5.1	19.2
Glucosyl rhamnosyl quercetin****	0.0	0.0	0.0	0.0	0.0	6.0
Hernandine**	0.0	3.6	1.8	0.0	0.0	4.8
N-caffeoyl tyramine**	38.0	8.5	2.6	0.0	103.9	35.8
Azelaic acid******	15.0	35.5	1.3	18.8	16.9	34.0
N-feruloyl tyramine**	0.0	0.0	0.0	0.0	0.0	2.4
N-feruloyl- 3'''-methoxy tyramine**	1.5	0.0	0.0	2.9	0.0	0.0

Biphenyl salsonoid B*	0.0	0.0	0.0	0.0	4.8	3.5
Hesperidin****	0.0	0.0	0.0	0.0	9.3	13.5
Tachioside*	0.0	4.8	0.0	0.0	0.0	0.0
Salsotetragonin*****	0.0	0.0	0.0	0.0	5.0	0.0
Glucopyranosyl oleanolic acid*****	0.0	12.4	0.0	0.0	0.0	10.8
Diosmin****	0.0	0.0	0.0	0.0	0.0	3.3
Salsamine**	0.0	0.0	0.0	0.0	0.0	0.0
Salsoloside D*****	80.2	131.4	82.0	26.8	3.5	12.9
Salsoloside E*****	22.0	36.8	42.4	9.6	0.0	0.0
Pentahydroxy-oleanen-oic acid glucopyranoside*****	0.0	4.4	0.0	0.0	0.0	0.0
Daphnoretin***	0.0	3.1	0.0	0.0	0.0	0.0
p-Coumaramide*	3.5	0.0	0.0	0.0	0.0	0.0
Salsoloside C*****	0.0	5.9	0.0	0.0	0.0	0.0
Salsolin A*****	0.0	0.0	0.0	0.0	0.0	6.7
Bergaptol***	0.0	0.0	0.0	0.0	0.0	4.2
Catechin****	0.0	0.0	0.0	12.1	0.0	23.2
Uridine**	2.1	0.0	0.0	0.0	0.0	0.0
Rosmarinic acid*	3.5	0.0	0.0	0.0	0.0	0.0
Fraxetin***	0.0	0.0	0.0	0.0	0.0	7.7
Trimethoxymethylene dioxy isoflavone****	0.0	0.0	0.0	0.0	11.1	0.0
Trihydroxy decosan trienoic acid******	0.0	0.0	0.0	0.0	19.9	0.0
Kaempferol****	0.0	0.0	0.0	4.2	29.2	4.5
Phenyl ethyl -gluco-Pyranoside*	76.3	44.3	28.5	45.7	12.9	19.2
Isofraxidin***	0.0	0.0	0.0	0.0	0.0	20.1
olean--en-diol*****	0.0	0.0	0.0	0.0	0.0	5.2
Scopoletin glucoside***	0.0	0.0	0.0	0.0	6.5	0.0
Naringenin****	0.0	0.0	0.0	0.0	4.9	0.0
Salsolains A*	0.0	0.0	0.0	0.0	16.3	0.0
Salsolic acid*****	13.5	9.9	7.3	7.3	0.0	0.0
Blumenyl Glucopyranoside*	0.0	0.0	0.0	0.0	5.3	0.0
Cuneataside C*	0.0	0.0	0.0	0.0	0.0	13.7
Staphylinoside D*	0.0	0.0	0.0	0.0	9.2	0.0
Hydroxy pseudoguai	0.0	0.0	0.0	0.0	0.0	5.7

en-olide; didehydro ketone*						
Canthoside D*	9.3	0.0	0.0	0.0	95.9	0.0
Lupeol*****	0.0	0.0	0.0	0.0	14.3	0.0
Gengirol*	16.1	48.6	11.1	19.5	21.1	15.6
Oxo-ionol-glucopyranoside*	0.0	0.0	0.0	0.0	0.0	5.7
Sitostanol*****	0.0	0.0	0.0	0.0	6.1	4.9
Hydroxy octadecadien-oic acid******	103.1	0.0	0.0	0.0	0.0	29.5
Phytol*	8.3	36.4	4.8	43.0	6.5	14.2
Salisoflavan****	0.0	6.2	0.0	6.4	0.0	0.0
Medicagenic acid*****	8.3	0.0	0.0	0.0	0.0	0.0
Calactin*****	6.8	0.0	0.0	0.0	0.0	0.0
Cyanidin****	0.0	0.0	0.0	0.0	0.0	14.4
Monolinolenin******	0.0	0.0	0.0	0.0	18.4	0.0
Oleanolic acid*****	0.0	0.0	0.0	0.0	7.4	0.0
Eicosenoic acid******	0.0	0.0	0.0	0.0	0.0	88.7
Apigenin****	30.5	0.0	0.0	0.0	0.0	0.0
Margaric acid******	0.0	6.7	0.0	0.0	8.2	123.8
Linoleic acid******	0.0	0.0	0.0	0.0	3.7	0.0
Umbelliferone***	0.0	0.0	0.0	0.0	0.0	13.0
Isorhamentin****	0.0	0.0	0.0	0.0	0.0	4.6
kaempferol-methyl ether****	15.2	0.0	0.0	0.0	0.0	0.0
Behenic acid******	0.0	0.0	0.0	0.0	32.8	18.2
Pinocembrin chalcone ****	5.4	0.0	0.0	0.0	0.0	0.0
Lignoceric acid******	0.0	0.0	0.0	0.0	0.0	5.3
Hexacosanoic acid ******	0.0	0.0	0.0	0.0	76.0	0.0
Octacosanoic acid ******	0.0	0.0	0.0	0.0	8.4	0.0

N.B Each standard of certain chemical class was used to calculate the relative concentration of the compounds of the same chemcial class by dividing the peak area of the compond over the peak area of the standard then utilizing slope and intercept of each standard, the concentarion of each metabolite of the same chemical class was calculated as mg/ equivalent then the value was divided by the amount of the total extract which is 20 mg (amount used in UHPLC study from each extract), the value would be mg equivalent (Eq.)/ g dry weight dry extractData are expressed as the average of five determinations (n=5)

* Compounds expressed as caffeic acid equivalent
** Compounds expressed as tyramine equivalent
*** Compounds expressed as scopoletin glycoside equivalent
**** Compounds expressed as hesperidin equivalent
***** Compounds expressed as oleanolic acid equivalent
****** Compounds expressed as azalaic acid equivalent

Compound	STA S. Tetrandra aerial aerial	STR S.Tetrandra root root	SVA S. Vermiculata aerial aerial	SVR S. Vermiculata root root	SGA S.Tetragona aerial aerial	SGR S.Tetragona root root
Trehalose	0	0	0	0	0	6934433
D-galacturonic acid	33538710	26665853	17697817	25115091.3	38109960	6832140
betaine	2.26E+08	7.85E+08	4.49E+08	370603504	2.24E+08	2.52E+08
Hypogallic acid	0	47700000	21900000	0	0	0
pericampylinone	0	6123503	0	0	0	0
cleomiscosin B	0	0	0	0	17700000	0
orsellic acid	8293654	0	0	0	0	0
caffeic acid	9808688	0	0	0	0	0
Anisic acid	0	7856925	0	0	0	0
Dirhamnosyl quercetin	66200000	0	0	0	0	0
biphenyl salsinol	0	7261220	6646399	0	0	0
N-(methoxy cinnamoyl)-norepinephrine	12500000	0	5149554	0	0	12100000
O-methyl gallic acid	0	0	18769819	0	0	0
feruloyloctopamine	1.28E+08	21200000	19169705	8059128.17	3.52E+08	1.08E+08
taxiphyllin	26577440	0	10661327	0	32030833	1.18E+08
hydroxy moupinamide	0	0	0	0	10115456	38353843
Glu-rham quercetin	0	0	0	0	0	12000000
hernandine	0	7254493	3684871	0	0	9635688
N-caffoyl tyramine	76100000	16944278	5118160	0	2.08E+08	71700000
Azelaic acid	30019564	71000000	26264868	37572861.8	33846928	67963843
feroulyl tyramine	0	0	0	0	0	4702353
N-feruloyl- 3'''-methoxy tyramine	2961579	0	0	5723660.92	0	0
Biphenyl salsonoid B	0	0	0	0	9589657	7009238
hesperidin	0	0	0	0	18500000	27000000
Isotachioside	0	9653235	0	0	0	0

salsotetragonin	0	0	0	0	9942323	0
glucopyranosyl oleanolic acid	0	24800000	0	0	0	21600000
Diosmin	0	0	0	0	0	6676919
salsamine	0	0	0	0	0	0
salsoloside D	1.6E+08	2.63E+08	1.64E+08	53623168.8	6960544	25800000
salsoloside E	44000000	73700000	84900000	19300000	0	0
Pentahydroxy-oleanen-oic acid;D-Glucopyranoside	0	8786581	0	0	0	0
Daphnoretin	0	6241628	0	0	0	0
p-Coumaramide	6904327	0	0	0	0	0
salsoloside C	0	11800000	0	0	0	0
salsolin A	0	0	0	0	0	13500000
Bergaptol	0	0	0	0	0	8329344
Catechin	0	0	0	24100000	0	46300000
uridine	4173434	0	0	0	0	0
Rosmarinic acid	0	0	0	0	50200000	11900000
Fraxetin	0	0	0	0	0	15449547
trimethoxy-methylenedioxy isoflavone	0	0	0	0	22200000	0
trihydroxy decosan-trienoic acid	0	0	0	0	39900000	0
trihydroxy-dimethoxy isoflavone	0	0	0	0	0	15520945
kaempferol	0	0	0	8374388.63	58300000	8973021
dihydroxy-phenyl-ethyl-D-glucopyranoside	1.53E+08	88645906	57100000	91433368.8	25823601	38400000
IsoFraxidin	0	0	0	0	0	40197388
olean-en-diol	0	0	0	0	0	10400000
scopoletin glucoside	0	0	0	0	12900000	0
Naringenin	0	0	0	0	9850393	0
salsolains A	0	0	0	0	32600000	0
Salsolic acid	27100000	19900000	14700000	0	0	0
Blumenyl B-□-D-glucopyranoside	0	0	0	0	10600000	0

cuneataside C	0	0	0	0	0	27422825
staphylinoside D	0	0	0	0	18500000	0
canthoside D	18600000	0	0	0	1.92E+08	0
lupeol	0	0	0	0	28700000	0
gengirol	32112318	97282513	22186676	38939374.2	42204928	31281728
Oxo-ionol-D-glucopyranoside	0	0	0	0	0	11423074
sitostanol	0	0	0	0	12200000	9708594
hydroxy octadecadienoic acid	2.06E+08	0	0	392600000	0	59100000
phytol	16527417	72813000	9614671	86088200.5	13004619	28321551
salisoflavan	0	12312770	0	12793158.6	0	0
medicagenic acid	16700000	0	0	0	0	0
dihydroxy trimethoxy isoflavone	0	0	0	0	0	7920999
calactin	13700000	0	0	0	0	0
cyanidin	0	0	0	0	0	28743176
monolinolenin	0	0	0	0	36846220	0
oleanolic acid	0	0	0	0	14800000	0
Eicosenoic acid	0	0	0	0	0	1.77E+08
Apigenin	60905299	0	0	0	0	0
Margaric acid	0	13337112	0	14536106.9	16435379	2.48E+08
linoleic acid	0	0	0	6272600.97	7414876	0
umbelliferone	0	0	0	0	0	25982879
Isorhamentin	0	0	0	0	0	9257949
kaempferol-methyl ether	30400000	0	0	0	0	0
behenic acid	0	0	0	0	65700000	36500000
Pinocembrin chalcone	10800000	0	0	0	0	0
lignoceric acid	0	0	0	0	0	10531167
Hexacosanoic acid	0	0	0	0	1.52E+08	0

octacosanoic acid	0	0	0	0	7.880204	0

Table S2: Peak intensities of each identified metabolite in *Salsola* extracts

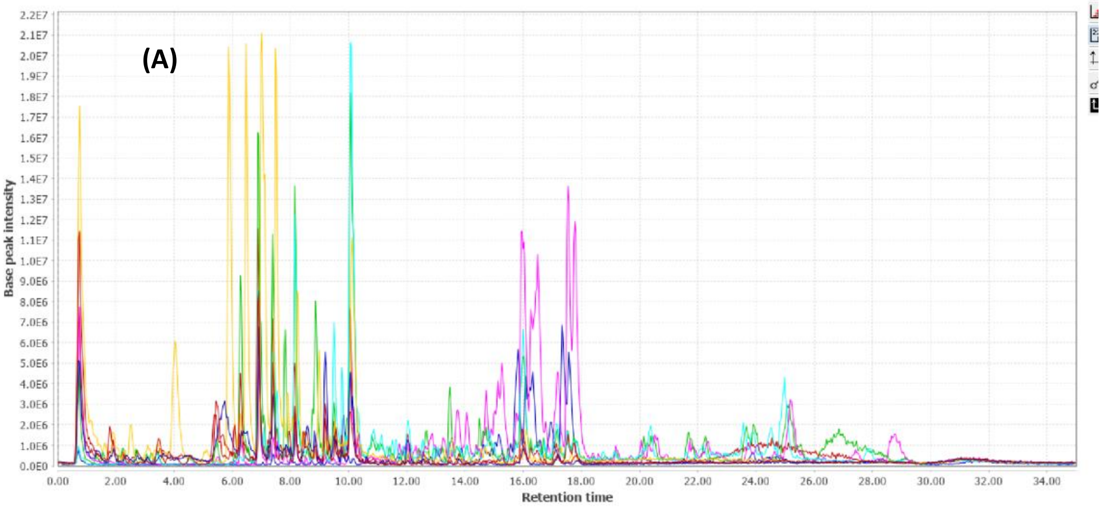

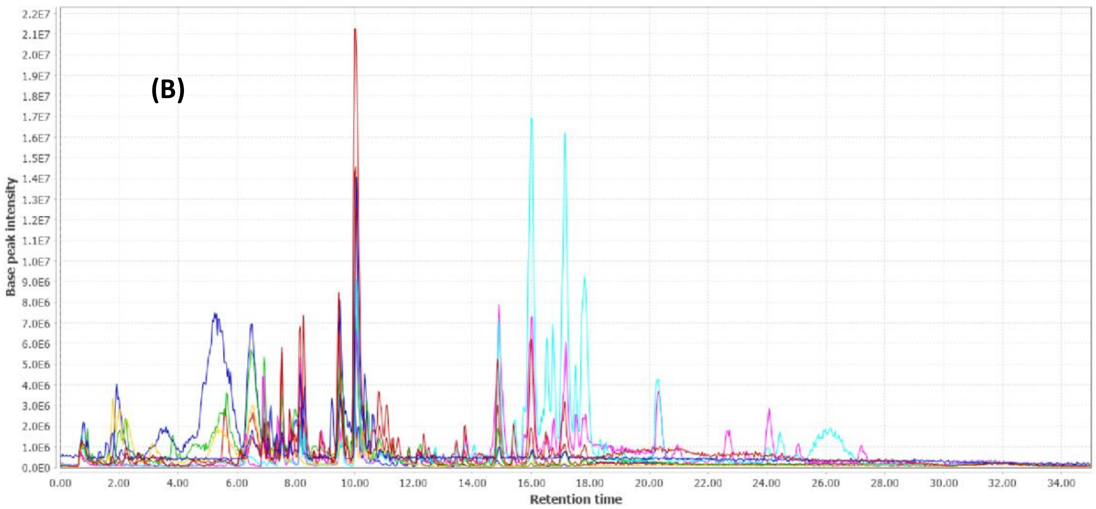

Figure S1: Base peak chromatogram of *Salsola* extracts in the negative ion mode (A) and positive ion mode (B)

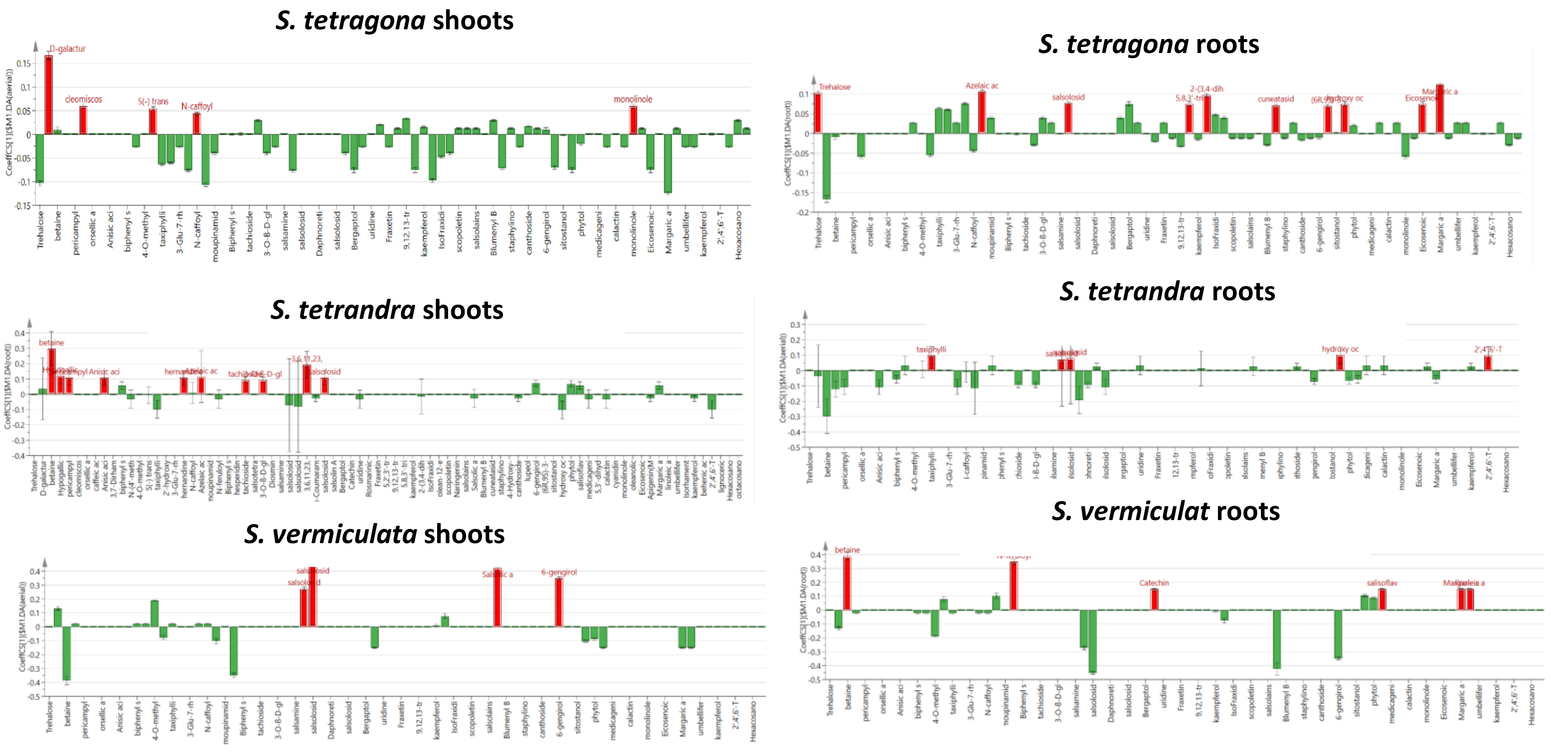

Figure S2: OPLS DA coefficients plots of *Salsola tetrandra*, *Salsola tetragona* and *Salsola vermiculata* shoots and root

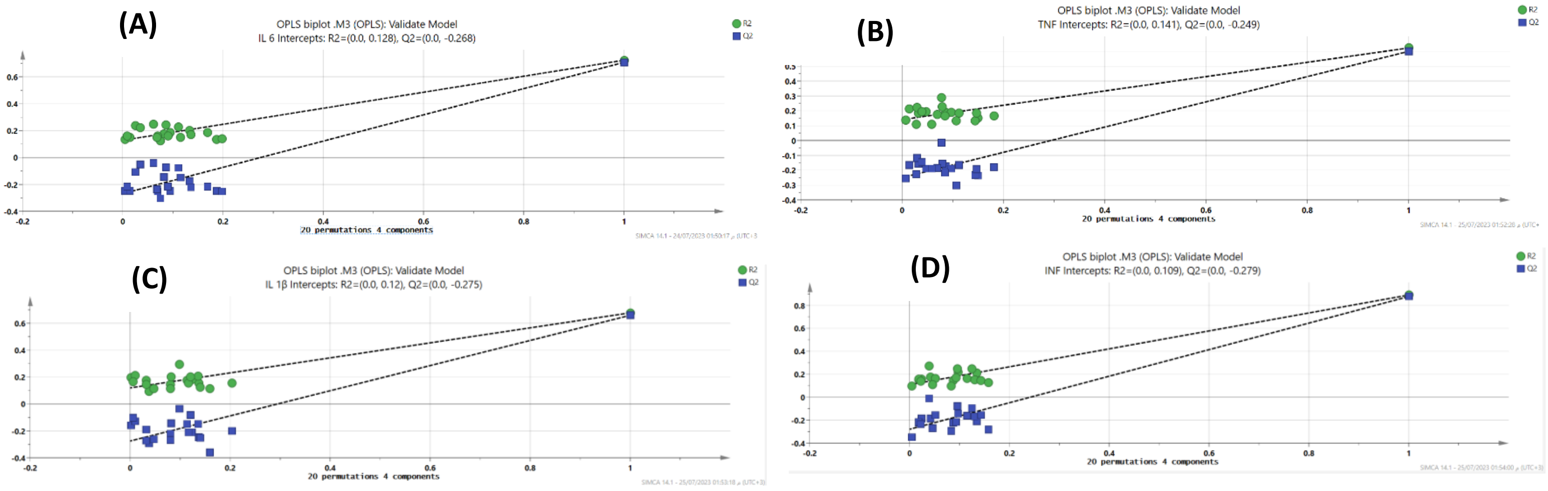

Figure S3: Permutation plots of IL 6 (A), TNF-α (B), IL 1*β* (C) and INF-γ (D)
